# Microsporidia Interact with Host Cell Mitochondria via Voltage-Dependent Anion Channels Using Sporoplasm Surface Protein 1

**DOI:** 10.1128/mBio.01944-19

**Published:** 2019-08-20

**Authors:** Bing Han, Yanfen Ma, Vincent Tu, Tadakimi Tomita, Joshua Mayoral, Tere Williams, Aline Horta, Huan Huang, Louis M. Weiss

**Affiliations:** a Department of Pathology, Albert Einstein College of Medicine, New York, USA; b Department of Medicine, Albert Einstein College of Medicine, New York, USA; Washington University School of Medicine; Tufts Medical Center; University of California—Davis

**Keywords:** *Encephalitozoon hellem*, microsporidia, mitochondria, parasitophorous vacuole, sporoplasm surface protein, voltage-dependent anion selective channels, VDAC

## Abstract

Microsporidia are opportunistic intracellular pathogens that can infect a wide variety of hosts ranging from invertebrates to vertebrates. During invasion, the microsporidian polar tube pushes into the host cell, creating a protective microenvironment, the invasion synapse, into which the sporoplasm extrudes. Within the synapse, the sporoplasm then invades the host cell, forming a parasitophorous vacuole (PV). Using a proteomic approach, we identified Encephalitozoon hellem sporoplasm surface protein 1 (EhSSP1), which localized to the surface of extruded sporoplasms. EhSSP1 was also found to interact with polar tube protein 4 (PTP4). Recombinant EhSSP1 (rEhSSP1) bound to human foreskin fibroblasts, and both anti-EhSSP1 and rEhSSP1 caused decreased levels of host cell invasion, suggesting that interaction of SSP1 with the host cell was involved in invasion. Coimmunoprecipitation (Co-IP) followed by proteomic analysis identified host cell voltage-dependent anion channels (VDACs) as EhSSP1 interacting proteins. Yeast two-hybrid assays demonstrated that EhSSP1 was able to interact with VDAC1, VDAC2, and VDAC3. rEhSSP1 colocalized with the host mitochondria which were associated with microsporidian PVs in infected cells. Transmission electron microscopy revealed that the outer mitochondrial membrane interacted with meronts and the PV membrane, mitochondria clustered around meronts, and the VDACs were concentrated at the interface of mitochondria and parasite. Knockdown of VDAC1, VDAC2, and VDAC3 in host cells resulted in significant decreases in the number and size of the PVs and a decrease in mitochondrial PV association. The interaction of EhSSP1 with VDAC probably plays an important part in energy acquisition by microsporidia via its role in the association of mitochondria with the PV.

## Abstract (precis)

Microsporidia are important opportunistic human pathogens in immune-suppressed individuals, such as those with HIV/AIDS and recipients of organ transplants. The sporoplasm is critical for establishing microsporidian infection. Despite the biological importance of this structure for transmission, there is limited information about its structure and composition that could be targeted for therapeutic intervention. Here, we identified a novel E. hellem sporoplasm surface protein, EhSSP1, and demonstrated that it can bind to host cell mitochondria via host VDAC. Our data strongly suggest that the interaction between SSP1 and VDAC is important for the association of mitochondria with the parasitophorous vacuole during microsporidian infection. In addition, binding of SSP1 to the host cell is associated with the final steps of invasion in the invasion synapse.

## INTRODUCTION

Microsporidia are obligate intracellular parasites that were initially identified about 150 years ago in studies on pébrine in silkworms (1). These pathogens can infect a wide variety of hosts ranging from invertebrates to vertebrates, including humans, and are widely distributed in nature, with over 200 genera and 1,400 species having been characterized. Among the species commonly infecting humans are Encephalitozoon cuniculi, Encephalitozoon hellem, and Encephalitozoon intestinalis, which infect both immunocompetent and immune-deficient hosts (2–6). These pathogens emerged as important opportunistic pathogens of humans with HIV/AIDS but are now recognized as widespread human pathogens, having been identified in recipients of organ transplants, the elderly, and children and in ocular infections in immunocompetent hosts (7, 8). Phylogenetic studies suggest that microsporidia are related to the fungi, probably as a sister group along with the Cryptomycota (9–11).

Microsporidia possess a unique, highly specialized invasion mechanism that involves the spore wall (SW), polar tube, and infectious sporoplasm. The spore (Sp) wall consists of proteins and chitin that protect the organism from harsh environmental conditions, thereby permitting transmission of the organism via water or food (12). The spore wall contains two layers: an electron-dense outer exospore layer and an electron-lucent inner endospore layer. Chitin is the main component of the endospore layer and is important for maintaining spore structure. Several spore wall proteins play crucial roles in spore adherence, signaling, and host cell interactions during the infection (13–17). The polar tube is a highly specialized invasion organelle of the microsporidia that coils around the sporoplasm inside the spore before germination (18, 19). Five polar tube proteins (polar tube protein 1 [PTP1] through PTP5) have been identified, among which PTP1 and PTP4 interact with host cells via mannose binding receptors and transferrin receptor 1 (TfR1), respectively, thereby enabling the polar tube to bind to the host cell surface and create an invasion synapse in which the sporoplasm can penetrate into the host cell (20–25). The infectious sporoplasm enters the host cell and undergoes development from meronts (proliferative forms) to sporonts, sporoblasts (Sb), and, finally, mature spores (6, 12, 26). Following entrance of the sporoplasm into the host cell, microsporidia (depending on the species) can reside directly in the host cell cytosol without additional barriers or can be found in a parasitophorous vacuole during various stages of the replicative cycle. Members of the genus *Encephalitozoon* spend their entire host cell cycle inside a PV (27–29). Ultrastructural analysis showed that the plasma membrane of meronts was closely associated with the PV membrane, and it has been suggested that they interact directly with the PV membrane (30–32). Meronts (Me) then detach from the PV membrane at a later stage of development, and maturing spores move to the center of the vacuole (32).

In the adaptation process that resulted in their becoming intracellular parasites, microsporidia underwent extreme genomic reduction and are missing genes for many metabolic pathways, including those for the biosynthesis of ATP (33, 34). They have a relic mitochondrion termed a mitosome, which lacks its own genome and has retained only a few metabolic functions but does not produce ATP (35, 36). This means that actively dividing microsporidia rely on their host cells as an energy resource (33). A previous study using *Encephalitozoon-*infected cells demonstrated that host mitochondria reorganize around the parasitophorous vacuole membrane (PVM), which was thought to be the strategy for microsporidia to obtain ATP from their host cell by maximizing contact between host mitochondria and the PV (33, 37, 38). This association of mitochondria with the PV starts early in infection, as soon as the PV forms after invasion (37). Transmission electron microscopy (TEM) showed that E. cuniculi uses electron-dense proteinaceous structures to tether mitochondria. This direct interaction between mitochondria and PVM was insensitive to the microtubule depolymerizing drugs albendazole and demecolcine, but was sensitive to protease treatment, suggesting that the relationship between mitochondria and the PVM is maintained by a protein-protein interaction (37, 38). The mitochondrial porin VDAC was demonstrated previously to concentrate at sites of contact between the microsporidian PVM and the host mitochondria, suggesting that it is involved in the microsporidian PV interaction with host mitochondria (38).

Other intracellular parasites have been reported to manipulate their host cells to facilitate invasion and growth. Due to the critical role of host cell mitochondria in metabolism and energy production, reorganization of mitochondria to the PVM has been described for several other obligate intracellular parasites, including the bacteria Legionella pneumophila and Chlamydia psittaci and the apicomplexan Toxoplasma gondii (39–42). In the case of T. gondii, parasite-secreted mitochondrial association factor 1 (MAF1), which localizes at the PV membrane, has been shown to play an important role in recruiting mitochondria during infection (43). Studies demonstrated that host mitochondrion association is mediated by an interaction between T. gondii MAF1 and the host cell mitochondrial intermembrane space-bridging (MIB) complex (44). In this paper, we identify and characterize a novel E. hellem sporoplasm surface protein, E. hellem sporoplasm surface protein 1 (EhSSP1), found in proteomic investigations of proteins involved in invasion. In addition to its involvement in invasion, EhSSP1 was demonstrated to interact with VDAC, which has been previously shown to be concentrated at sites of contact between the microsporidian PVM and the host mitochondria (38).

## RESULTS

### Identification of E. hellem sporoplasm protein 1 (EhSSP1).

**FIG 1:**
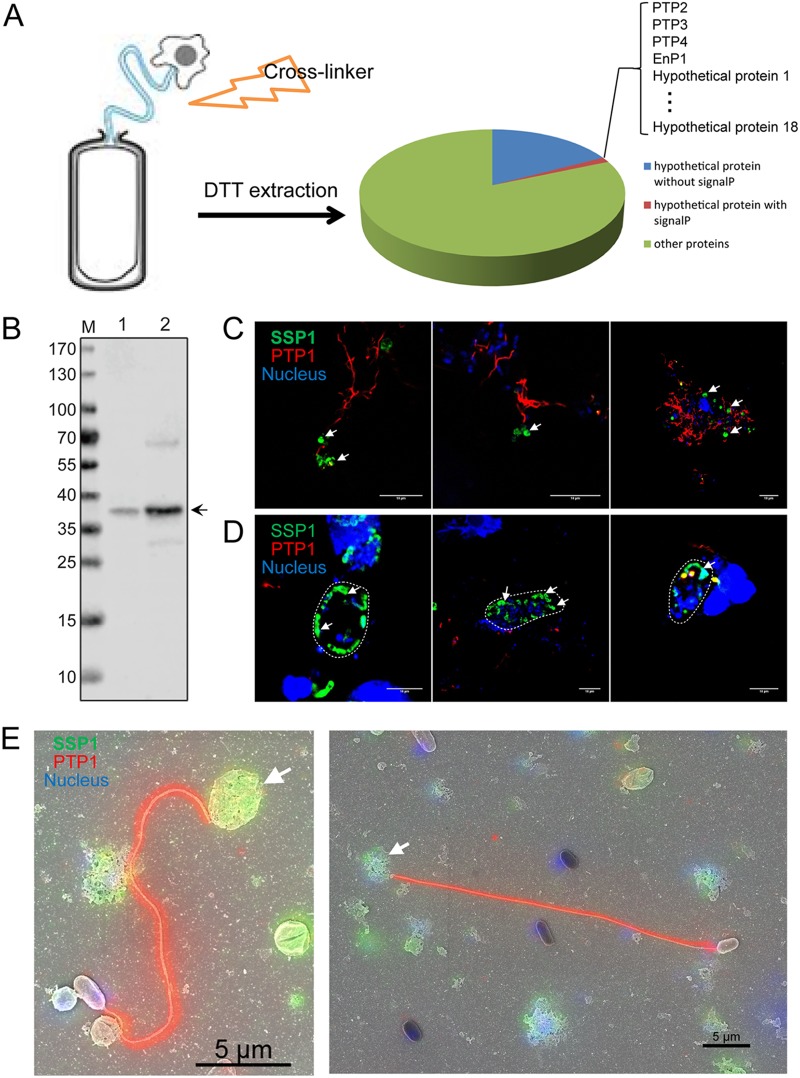
Characterization of E. hellem sporoplasm surface protein 1 (EhSSP1). (A) Schematic showing the proteomic analysis pipeline. Spores were lysed in SDS buffer, and the pellet was treated with cross-linker reagent and extracted with DTT. The DTT extraction was then analyzed with LC-MS/MS, and 22 hypothetical proteins were identified. (B) Immunoblot detection of native SSP1 in DTT extractions (lane 1) and spore lysis (lane 2). The mouse polyclonal antibody was used at a 1:100 dilution. M, molecular mass marker lane. (C) Extruded sporoplasms of E. hellem were initially incubated with anti-EhSSP1 mPcAb at a 1:100 dilution and detected by anti-mouse Alexa Fluor 488 secondary antibody (green). The polar tubes were stained with anti-PTP1 rPcAb and detected by anti-rabbit Alexa Fluor 594 secondary antibody. White arrows indicate the labeling of EhSSP1 on a sporoplasm at the tips of the polar tubes. Bar, 10 μm. (D) A productive meront stage was specifically stained by anti-EhSSP1 mPcAb at the periphery of the PVM (white line). Bar, 10 μm. (E) CLEM analysis of the localization of EhSSP1 further proved that it was located on the sporoplasm (white arrows), Bar, 5 μm.

EhSSP1 (EHEL_111090) was identified in a proteomic screen of our previously described PTP preparation of polar tubes (23) that was modified by using a cross-linker strategy as described in Materials and Methods (see below). During germination of spores, the sporoplasm is transported to the host cell via the polar tube (45, 46), so a cross-linker was used to help find proteins that bound to the polar tube during germination. It was probable that some polar tube proteins (PTPs) interacted with sporoplasm proteins (SPs) during this process. The cross-linked complex was extracted by the use of dithiothreitol (DTT) and analyzed by liquid chromatography-tandem mass spectrometry (LC-MS/MS) (Fig. 1A). There were about 300 genes identified from this proteomic analysis which were predicted as unknown hypothetical proteins in MicrosporidiaDB (https://microsporidiadb.org/micro/). To narrow our search to identify surface proteins (and also new polar tube proteins), we examined these hypothetical proteins to identify those with a predicted signal peptide. This resulted in the identification of a subset of 22 proteins from E. hellem (see Table S1 in the supplemental material). Four of these were demonstrated by BLAST to be homologous to PTP2, PTP3, PTP4, and EnP1, all of which had been identified previously in various microsporidia, confirming the utility of this approach for identifying components of the polar tube and spore coat (22, 23, 47). To examine the other 18 proteins, these proteins were expressed as recombinant proteins in Escherichia coli and purified for immunization of mice, and the resulting mouse polyclonal antibodies (mPcAb) were used to determine the subcellular localization of each protein by indirect immunofluorescence assay (IFA) in E. hellem*-*infected host cells. On the basis of the IFA results, EHEL_111090 was selected for further study and was renamed E. hellem sporoplasm surface protein 1 (EhSSP1).

10.1128/mBio.01944-19.6 TABLE S1 Hypothetical proteins identified from LC-MS/MS analysis. Download Table S1, DOCX file, 0.01 MB. Copyright © 2019 Han et al. 2019 Han et al. This content is distributed under the terms of the Creative Commons Attribution 4.0 International license.

By IFA, EhSSP1 was found to localize to the germinated sporoplasm at the tip of polar tube (Fig. 1C) and to microsporidian vegetative meronts in the periphery of the parasitophorous vacuole (PV) (Fig. 1D). There was no staining of extracellular germinated spores (Fig. 1C) or mature spores in the middle of PV (Fig. 1D). Immunoblotting using mouse polyclonal antibody (mPcAb) and rabbit polyclonal antibody (rPcAb) to EhSSP1 demonstrated that it was present in both the DTT extraction preparation and the spore total lysate extracted by SDS and that it had a molecular mass of ∼37 kDa (Fig. 1B; see also Fig. S2 in the supplemental material), which is slightly greater than the predicted molecular mass of 33 kDa.

10.1128/mBio.01944-19.2 FIG S2 (A) Coomassie brilliant blue staining of purified rEhSSP1 subjected to SDS-PAGE. (B) Immunoblot detection of native EhSSP1 using rabbit anti-EhSSP1 polyclonal antibody at a 1:500 dilution. Download FIG S2, TIF file, 1.9 MB. Copyright © 2019 Han et al. 2019 Han et al. This content is distributed under the terms of the Creative Commons Attribution 4.0 International license.

Correlative fluorescence and scanning electron microscopy (CLEM) techniques were used to further confirm the location of EhSSP1. As shown in Fig. 1E, the CLEM data clearly demonstrated localization of EhSSP1 to the germinated sporoplasm at the tip of polar tube, confirming that this is a novel sporoplasm protein.

### EhSSP1 is a sporoplasm surface protein and is exposed to the host cell during the early stages of microsporidian infection.

**FIG 2:**
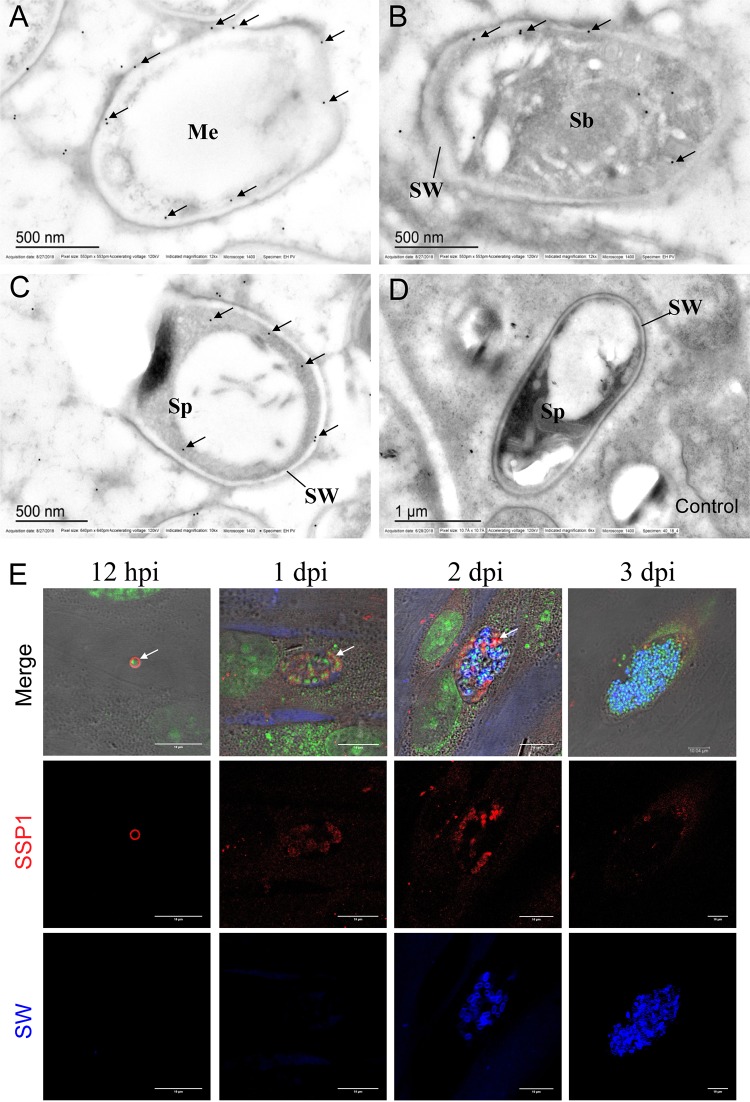
EhSSP1 is a sporoplasm surface protein that is exposed to the host cell during infection. (A to D) Immuno-EM shows the localization of EhSSP1 in E. hellem spores. Native SSP1 was located on the surface of the productive meront stage when the spore wall had not formed (A), and when the spore wall was partially formed in the sporoblast (B) or after it was fully formed in mature spores (C), the gold particles localized to the interface of spore wall and sporoplasm, further demonstrating that EhSSP1 is a sporoplasm membrane surface protein. (D) Negative control. Bar, 500 nm (A to C) or 1 μm (D). (E) Immunofluorescence assay (IFA) detection of native EhSSP1 in E. hellem within HFF cells showing that EhSSP1 is exposed to the host cell during infection. The sporoplasm membrane was stained by anti-EhSSP1 mPcAb (red) when the sporoplasm had just transformed into host cell cytoplasm, at 12 hpi; anti-EhSSP1 stained meronts at the edge of PVM at 1 dpi and 2 dpi. Spores with a spore wall are seen in the middle of PV and were stained with Fluorescent Brightener (calcofluor white [blue]) at later stages of infection 3 dpi where the PV was filled with mature spores and there was no EhSSP1 staining in the vacuole. Nuclei were stained with SYTO green fluorescent nucleic acid stain. Bar, 10 μm.

To further investigate the ultrastructure location of EhSSP1, immunoelectron microscopy (immuno-EM) was conducted using anti-EhSSP1 mPcAb. The E. hellem developmental stages reacting with anti-EhSSP1 in the parasitophorous vacuole are shown in Fig. 2. Gold particles were observed on the surface of meront (Me) stages, which do not have a spore wall (Fig. 2A). This confirmed that that EhSSP1 was exposed on the surface of this early stage of microsporidia. In the sporoblasts (Sb), which have a partially formed spore wall, and in the mature spore (Sp), in which the spore wall is fully formed, gold particles were located beneath the spore wall at the interface of the spore wall and sporoplasm membrane (Fig. 2B and C). This further confirmed that EhSSP1 locates on the surface of microsporidian sporoplasm and appears to be a plasma membrane-associated protein. There was no staining seen when a control mouse serum was used (Fig. 2D).

A time course infection assay was then conducted to examine the staining of EhSSP1 during development of the parasitophorous vacuole. The host cell was fixed from early after 12 h postinfection (hpi) through a later stage at 3 days postinfection (dpi). Cells were stained with anti-EhSSP1 mPcAb (red fluorescence), and mature spores, which had formed the spore wall, were stained with the fluorescence brightener (FB [blue florescence]) (Fig. 2E). These IFA results demonstrated that anti-EhSSP1 had stained the germinated sporoplasm in a ring pattern at 12 hpi, which is consistent with the immuno-EM data presented in Fig. 2A. This suggested that EhSSP1, as a surface protein, would be exposed to the host cell after the sporoplasm was transported to the host by the polar tube. There were no spores stained with the fluorescence brightener at 1 dpi, revealing that the spore wall was not yet present at that early time point and that the microsporidia in the PV that had been stained by anti-EhSSP1 were likely meronts (the proliferative forms). Some of the spores were seen to have been stained with the fluorescence brightener at 2 dpi, and these were mainly located in the middle of the PV. There was no EhSSP1 staining of these spores, probably because EhSSP1 was not accessible to the antibody when it was beneath the spore wall. The meronts at the edge of PV still could be stained by anti-EhSSP1 at 2 dpi. When the infection progressed to 3 dpi, all of the spores stained with the fluorescence brightener and there was minimal to no staining by antiEhSSP1 in the PV. These data suggest that EhSSP1 is exposed at the stage during which the parasites are contacting host cells either in the host cytoplasm or at the interface of PV membrane, revealing a potential role for this protein in interactions between the microsporidia and the host cell.

### rEhSSP1 can bind to the polar tube by interacting with polar tube protein 4 (PTP4).

**FIG 3:**
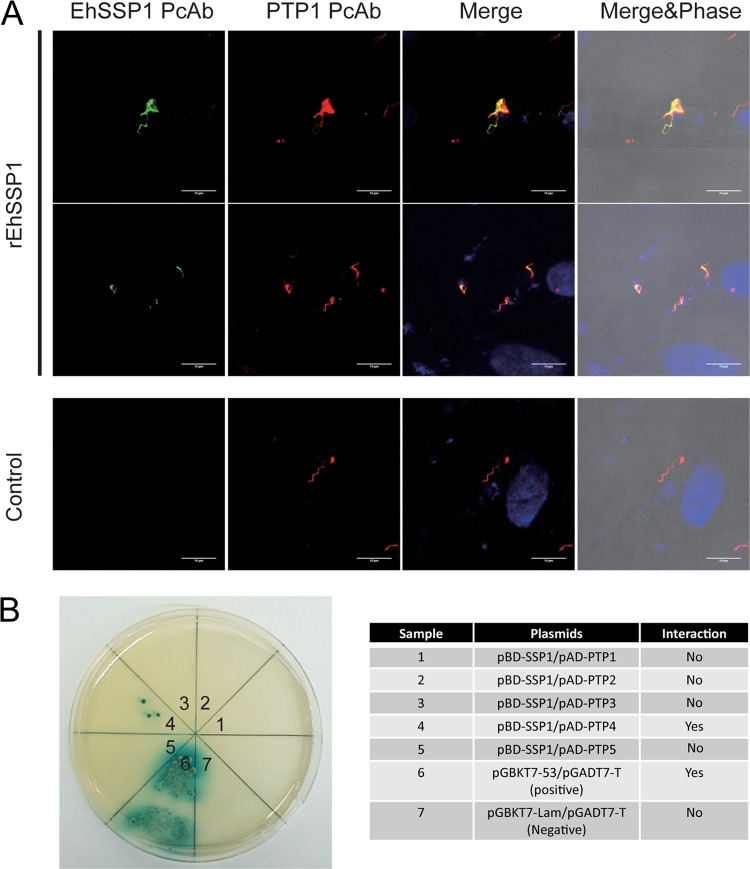
EhSSP1 can bind to the polar tube by interacting with polar tube protein 4. (A) Immunofluorescence microscopy (IFA) of rEhSSP1 binding to the polar tube. The rEhSSP1 was incubated with germinated spores in infected cell cultures and stained with anti-EhSSP1 mPcAb (green), while the polar tube was stained with anti-EcPTP1 PcAb (red). The merged panel demonstrates that rEhSSP1 was able to bind extruded polar tubes. (B) Yeast two-hybrid analysis of EhSSP1 interactions with polar tube proteins. This illustrates the interactions of the pAD and pBD (bait and prey) constructs containing full-length EhPTP1 to EhPTP5 (bait) and EhSSP1 (prey). Results of the positive-control (pGBKT7-53/pGADT7-T) and negative-control (pGBKT7-Lam/pGADT7-T) reactions are shown. The results show that EhSSP1 interacted with EhPTP4 on the polar tube.

EhSSP1 was identified from the cross-linked DTT preparation, so we examined whether it could interact with any of the known polar tube proteins (PTPs) and whether it might be involved in polar tube interactions with the sporoplasm. We used both a protein binding assay and a yeast two-hybrid (Y2H) method to investigate this interaction. To evaluate protein binding directly, we germinated the spores in the cell culture, and recombinant EhSSP1 protein (rEhSSP1) was incubated with the germinated polar tubes. As demonstrated in Fig. 3A (see also Fig. S3), rEhSSP1 (identified by antiEhSSP1 mPcAb) did bind to the polar tube (identified by anti-PTP1 PcAb [48]), suggesting that native EhSSP1 also would bind to this structure. A yeast two-hybrid method was conducted to assess the specificity of binding of EhSSP1 to the various PTPs that have been identified. The coding regions of the five known polar tube proteins (PTP1 to PTP5) were used to construct pAD prey vectors, and EhSSP1 was used to construct a pBD bait vector. The transformed yeast strains were mated and selected on a QDO/X/A selection plate. As demonstrated in Fig. 3B, EhSSP1 could specially interact with polar tube protein 4 (PTP4), but not with the other polar tube proteins, suggesting that EhSSP1 could bind to polar tube via an interaction with PTP4. We previously reported that PTP4 can be found at the tip of the polar tube (23) within the invasion synapse. Thus, it is possible that binding of SSP1 to PTP4 during extrusion of the sporoplasm from the polar tube tip acts as an anchor for the sporoplasm onto the polar tube.

10.1128/mBio.01944-19.3 FIG S3 Immunofluorescence microscopy (IFA) of EhSSP1 binding to polar tube. rEhSSP1 was incubated with germinated spores in infected cell culture and stained with both antiEhSSP1 mPcAb (green) and anti-HA monoclonal antibody (MAb) (red). The merged panel demonstrates that rEhSSP1 can bind to extruded polar tubes. Bar, 10 μm. Download FIG S3, PDF file, 0.1 MB. Copyright © 2019 Han et al. 2019 Han et al. This content is distributed under the terms of the Creative Commons Attribution 4.0 International license.

### rEhSSP1 can interact with host cells and plays a role during infections.

**FIG 4:**
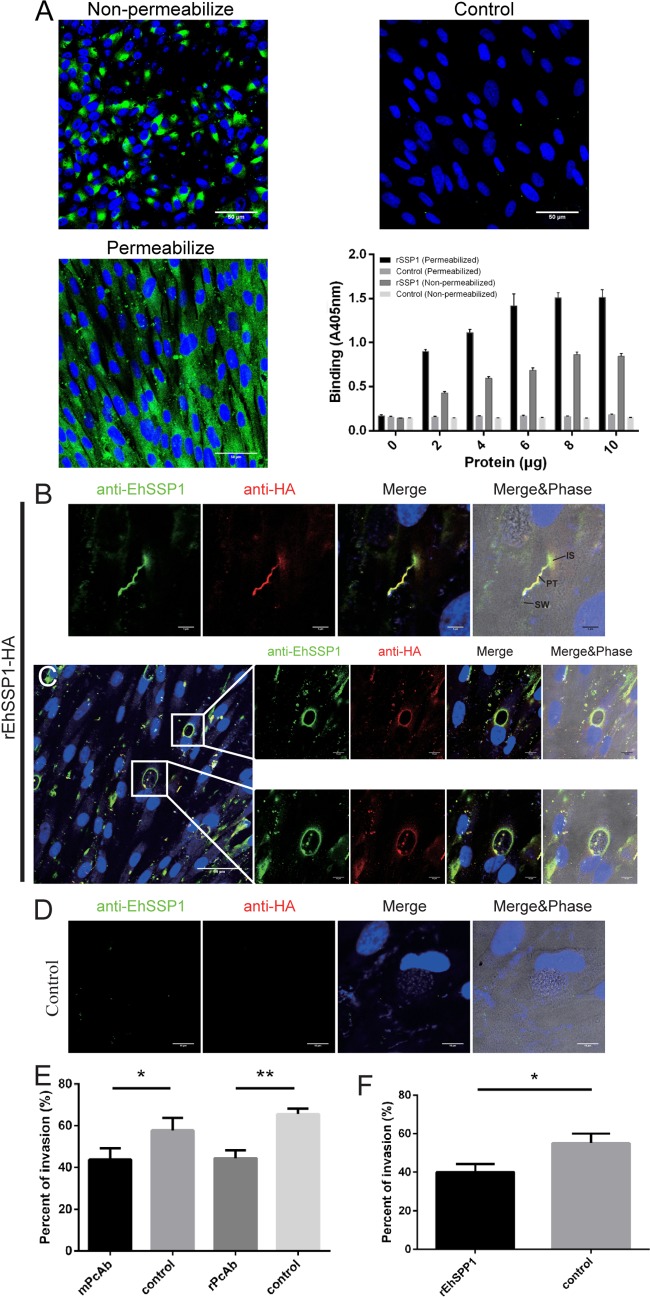
EhSSP1 can interact with host cells. (A) Binding of rEhSSP1 detected by IFA and ELISA. HFF cells were grown on glass coverslips and treated with or without permeabilization buffer followed by incubation with rEhSSP1. Binding was detected using fluorescent goat anti-mouse secondary antibody. Different concentrations of rEhSSP1 were incubated with permeabilized or nonpermeabilized HFF cells in 96-well plates, and binding was detected via ELISA. BSA was used at the same concentration as EhSSP1 as a negative control in both IFA and ELISA. All experiments were performed in triplicate. Data are presented as means ± standard deviations (SD). (B) By IFA, both anti-EhSSP1 PcAb (green) and anti-HA antibody (red) show the binding of EhSSP1 to the polar tube (PT) and invasion synapse (IS) on the host cell surface. An empty spore wall is seen connecting with the polar tube. Bar, 5 μm. (C) IFA demonstrating that EhSSP1 can interact with the PV membrane in infected cells. Bar, 50 μm (left panel) or 10 μm (enlarged panels). (C) IFA negative control. Bar, 10 μm. (E and F) Interference with EhSSP1 host cell interactions alters microsporidian infectivity. (E) Impact of rabbit polyclonal anti-EhSSP1 (rabbit and mouse PcAb) on E. hellem infection of HFF cells. E. hellem spores were incubated with anti-EhSSP1 (∼5 μg/ml) for 1 h at room temperature and then added to HFF monolayers. Control organisms were treated with the same amount of seronegative rabbit or mouse serum as the negative control. Anti-EhSSP1 significantly reduced the PV number in infected HFF cells. mPcAb: *, *P* < 0.05. rPcAb: **, *P* < 0.01. (F) Impact of exogenous rEhSSP1 on E. hellem infection of HFF cells. Purified rEhSSP1 (5 μg/ml) was incubated with HFF cells for 1 h at room temperature, and the HFF were then infected with 1 × 10^6^ spores/well. The same amount of BSA (5 μg/ml) was used as a negative control. rEhSSP1 significantly reduced the PV number of HFF cells infected by E. hellem (*, *P* < 0.05).

As EhSSP1 is found on the surface of the sporoplasma and is exposed to the host cell in the invasion synapse, the interaction of EhSSP1 with host cells was evaluated. By IFA, rEhSSP1 was demonstrated to bind to both permeabilized and nonpermeabilized cells (Fig. 4A). An enzyme-linked immunosorbent assay (ELISA) also demonstrated that rEhSSP1 bound to host cells at higher levels in permeabilized cells than in nonpermeabilized cells (Fig. 4A). The IFA and ELISA data suggest that EhSSP1 interacts with a host cell protein(s) that is probably present both on the cell surface and within the host cell. The binding of rEhSSP1 to E. hellem*-*infected host cells was also evaluated (Fig. 4B and C). rEhSSP1 was found to bind to the germinated polar tube and the invasion synapse (IS) where the tip of polar tube attaches to host cells (Fig. 4B; see also Fig. S4), indicating that the sporoplasm may interact with the host cell via an interaction of EhSSP1 with a host cell target in the invasion synapse after spore germination during infection. In permeabilized cells, interestingly, rEhSSP1 also bound to the PVM (Fig. 4C; see also Fig. S5). This binding suggests that the presence of exposed EhSSP1 on the surface of meronts might be one of the reasons that the vegetative forms of microsporidia stay in contact with the PV membrane at the periphery of PV. Once the spore wall is formed, SSP1 would no longer be accessible for this interaction and this would be consistent with the location of mature spores within the interior of the PV. In the control experiments, no staining was seen with cells or on the PVM (Fig. 4D).

10.1128/mBio.01944-19.4 FIG S4 Immunofluorescence microscopy (IFA) of the binding of EhSSP1 to the polar tube and invasion synapse on the host cell surface stained by both the anti-EhSSP1 PcAb (green) and anti-HA antibody (red). An empty spore wall is seen connecting with the polar tube without any staining. Bar, 10 μm. Download FIG S4, PDF file, 0.2 MB. Copyright © 2019 Han et al. 2019 Han et al. This content is distributed under the terms of the Creative Commons Attribution 4.0 International license.

10.1128/mBio.01944-19.5 FIG S5 Immuno-EM detection of rEhSSP1 binding to the PVM. rEhSSP1 was incubated with ultrathin sections of infected cells on nickel grids and then stained with anti-EhSSP1 mPcAb at dilution of 1:50. The gold particles (black arrows) demonstrate binding of rEhSSP1 on the PVM. Bar, 5 μm (left panel) or 1,000 nm (enlarged panel). Download FIG S5, PDF file, 0.1 MB. Copyright © 2019 Han et al. 2019 Han et al. This content is distributed under the terms of the Creative Commons Attribution 4.0 International license.

Due to the interaction of EhSSP1 with host cells, the role of EhSSP1 during the microsporidian infection was investigated by antibody blocking and protein competition experiments. Anti-EhSSP1 rPcAb and anti-EhSSP1 mPcAb were incubated with purified spores for 1 h, and the mixture was added to the medium during host cell infection. The presence of antibody to EhSSP1 significantly reduced infectivity (Fig. 4E). In a similar fashion, the addition of rEhSSP1 directly to the cell culture prior to infection with spores also significantly decreased infection (Fig. 4F). Overall, this suggests that EhSSP1 interacts with the host cell during the early stages of infection and that the interaction likely occurs in the invasion synapse when the sporoplasm first comes into contact with the host cell surface.

### rEhSSP1 interacts with host VDAC.

**FIG 5:**
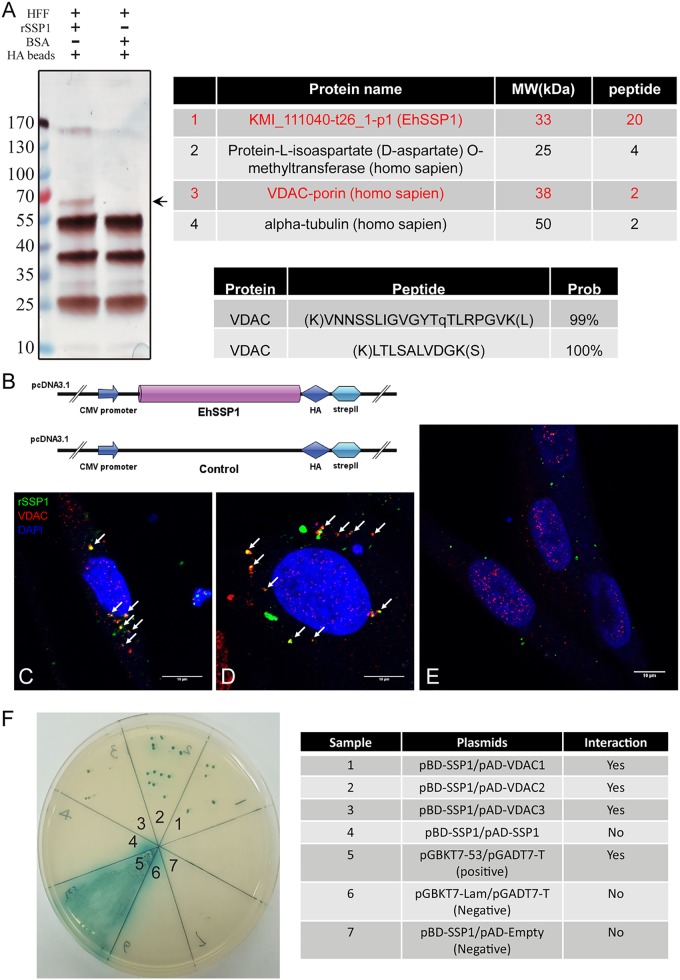
VDAC was identified as the interaction partner of EhSSP1. (A) Immunoprecipitation (IP) and LC-MS/MS identified VDAC as the interacting protein of EhSSP1. BSA was used as a negative control. The arrow indicates the band of interest in the silver-stained gel of the IP sample. The MS/MS spectrum identified two peptides of VDAC from IP samples. MW, molecular weight. (B) Diagrams representing the construction of the mammalian cell expression of EhSSP1 in pcDNA 3.1 vector and negative plasmid. (C and D) Confocal laser scanning microscopy of EhSSP1 (green) and VDAC (red) in two different HFF cells that express rEhSSP1. rEhSSP1 and native VDAC colocalized inside host cells (white arrows). Bar, 10 μm. (E) Staining of EhSSP1 and VDAC in HFF cells transfected with a negative construct where no colocalization signal was identified in the control panel. (F) Yeast two-hybrid analysis of EhSSP1 interactions with EhSSP1 itself and three isoforms of VDAC. This illustrates the interactions of the pAD and pBD (bait and prey) constructs containing full-length EhSSP1 and human VDAC1 to VDAC3. The results of the positive-control pGBKT753/pGADT7-T, negative-control pGBKT7-Lam/pGADT7-T, and negative-control pBD-EhSSP1/pAD-empty reactions are shown. The data demonstrate that EhSSP1 can interact with all three isoforms of VDAC but not does not interact with itself.

The host cell protein interacting with EhSSP1 was evaluated using a proteomic analysis of a pulldown with hemagglutinin (HA)-tagged rEhSSP1. Silver staining of the rEhSSP1-HA pulldown complexes revealed an extra protein band in the test lane compared to the control group (Fig. 5A). LC-MS/MS data identified 30 peptides from EhSSP1, confirming that the pulldown complex contained rEhSSP1, as expected. Several host cell proteins that might be the potential targets of EhSSP1 were identified (Fig. 5A). Among these, the most likely candidate was VDAC due to its location on both the plasma membrane and the outer mitochondrial membrane (OMM) (49, 50). Also, this would explain a role for EhSSP1 in the interaction of meronts with the PV membrane and in the interaction of the sporoplasm with the plasma cell membrane within the invasion synapse.

To verify an interaction between VDAC and EhSSP1, the coding sequence of EhSSP1 was cloned into a mammalian cell expression vector (Fig. 5B) and transfected into human foreskin fibroblasts (HFF). The locations of the expressed rEhSSP1 and native VDAC were examined by IFA using antibodies as shown in Fig. 5C and D, and the results demonstrated that rEhSSP1 colocalized with VDAC in the cell cytoplasm. A small amount of background signal was seen in the control group; however, no colocalization was observed (Fig. 5E).

A yeast two-hybrid assay was then conducted to further examine interactions of EhSSP1 and the three known human VDAC isoforms (hVDAC1, hVDAC2, and hVDAC3). As demonstrated in Fig. 5F, EhSSP1 interacted with all of the VDAC isoforms, but EhSSP1 did not interact with itself or with the empty prey vector (Fig. 5F).

### rEhSSP1 colocalizes to host mitochondria.

**FIG 6:**
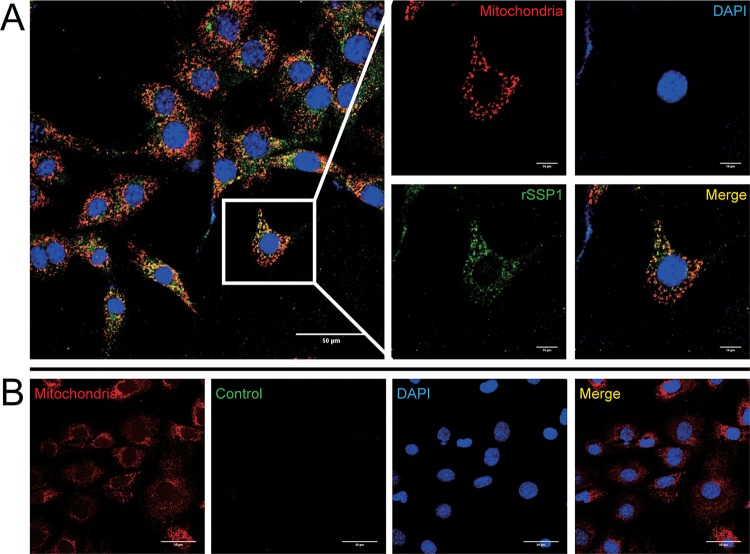
Confocal laser scanning analysis of EhSSP1 and mitochondria in MEFs. (A) The mitochondria of MEF cells were stained with 100 nM MitoTracker (Red) for 30 min. After fixation and permeabilization, the cells were blocked and incubated with rEhSSP1 (green). Nuclei were stained with DAPI (blue). The merged image demonstrates colocalization of rEhSSP1 and mitochondria in the cytoplasm of MEF cells. Bar, 50 μm (left panel) or 10 μm (enlarged panels). (B) The negative control (BSA) demonstrated no protein binding to mitochondria and an absence of a colocalization signal. Bar, 50 μm.

As VDAC is found on the outer mitochondrion membrane, we next investigated the binding of rEhSSP1 to host cell mitochondria in both infected and uninfected mouse embryonic fibroblasts (MEFs). Mitochondria were labeled by MitoTracker (red fluorescence), and the binding of rEhSSP1 was detected by antiEhSSP1 mPcAb (green fluorescence). Panel A of Fig. 6 shows that rEhSSP1 colocalized with the mitochondria in the host cell cytoplasm. This is consistent with EhSSP1 binding to mitochondria by an interaction with VDAC, and there was no colocalization seen in the negative control (Fig. 6B).

### Host mitochondria accumulate around the PV and interact with microsporidian meronts.

**FIG 7:**
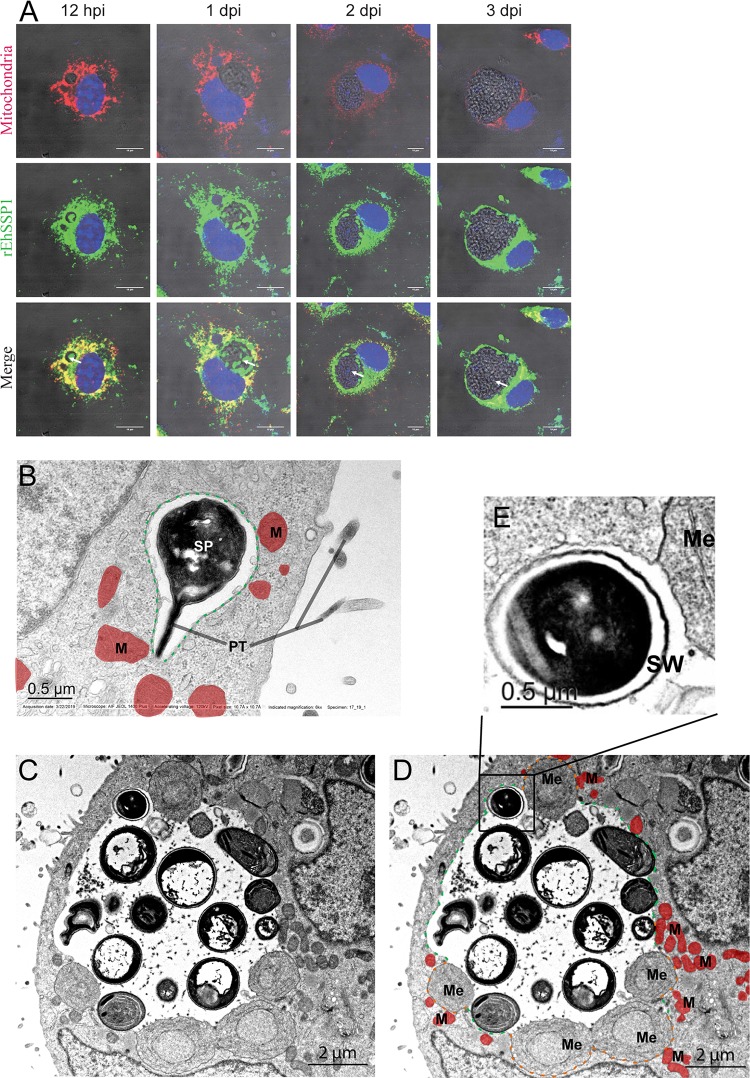
Host mitochondria accumulate around the PV and interact with microsporidian meronts. Fluorescent images of host mitochondrial PV interaction during the development of E. hellem in MEF cells. As E. hellem development progressed (12 hpi and 1, 2, and 3 dpi), the mitochondria (red) were seen to accumulate around the edge of the PV (white arrows) and rEhSSP1 (green) was seen to interact with these mitochondria at the PV membrane. Bar, 10 μm. (B) Electron micrograph of an MEF cell with an E. hellem sporoplasm (SP) inside a host-cell cytoplasmic PV (green outline). The presence of a segment of the polar tube (PT) still attached to the sporoplasm indicates it was the initial start of the infective process. Note the presence of mitochondria (M, dark red) abutting the edge of the forming PVM (green). Bar, 0.5 μm. (C and D) Transmission electron micrograph of an MEF cell with an E. hellem PV containing proliferative (meront) and sporogonic stages of parasite development. The proliferative stages (meronts) are enclosed by a “thin” membrane and are abutted to the periphery of the vacuole. Numerous host mitochondria (M, colored dark red in image D) are in close proximity to these early stages, and some appear to have formed an interconnected complex encompassing the PV-parasite-mitochondrion membranes. As the parasites enter sporogony, the membranes become thicker and more electron dense. This thickening continues and eventually results in formation of the thickened sporoblast (Sb) and spore wall. During sporogony, tubular projections formed on the parasite surface which eventually sloughed off and accumulated in the PV. Meronts (Me) were located at the edge of PVM (colored green in image D), and spores with spore walls detached from the PVM and moved to the middle of the PV. The meront surface was found in close association with the PVM, forming an electron-dense membrane structure (EDMS, colored orange in image D). Mitochondria clustered around the meronts at the periphery of PV. Bar, 2 μm. (E) The enlarged panel shows a spore in which the spore wall was forming as it was detaching from the PVM and moving to the middle of PV. Bar, 0.5 μm.

In the infected cells, as demonstrated in Fig. 7A, host mitochondria preferentially clustered around the PV, and this was more prevalent at the early stages of infection stage (1 dpi) than at the later infection stage (2 dpi and 3 dpi). This is consistent with predictions, because less EhSSP1 was exposed in mature PV life cycle stages, representing the time point at which the microsporidia develop a spore wall that would prevent an interaction between EhSSP1, found on the surface of meronts but beneath the spore wall in later stages, and VDAC on the surface of mitochondria surrounding the PV.

TEM performed at the early stage of infection, when the sporoplasm exiting the polar tube had invaded via the invasion synapse into the host cell, demonstrated the presence of mitochondria already clustering around the PV and that many of those mitochondria were in close contact with the PVM (Fig. 7B). This is consistent with the data seen at 12 hpi (Fig. 7A), which demonstrated that mitochondria had reoriented to the vicinity of the microsporidian PV at this very early stage of infection. At a later stage of infection, the meronts were found immediately beneath the PVM lining the vacuole, and mature spores were found in the middle of the PV (Fig. 7C). As revealed by TEM, the meronts bulged into cell cytoplasm, and in several images, the surface of the meronts appeared to merge with the PV membrane at the bulging areas, forming an electron-dense membrane structure (EDMS) (Fig. 7C and D). Mitochondria were clustered around the PV and were found in particular around the areas where the meronts bulged into cytoplasm (Fig. 7C and D). Panel E of Fig. 7 shows a more mature stage (an early spore) detaching from the PVM, presumably due to the spore wall blocking the interaction between EhSSP1 and the PVM.

### VDAC is found at the interacting interfaces between the host cell mitochondria, PVM, and meronts.

**FIG 8:**
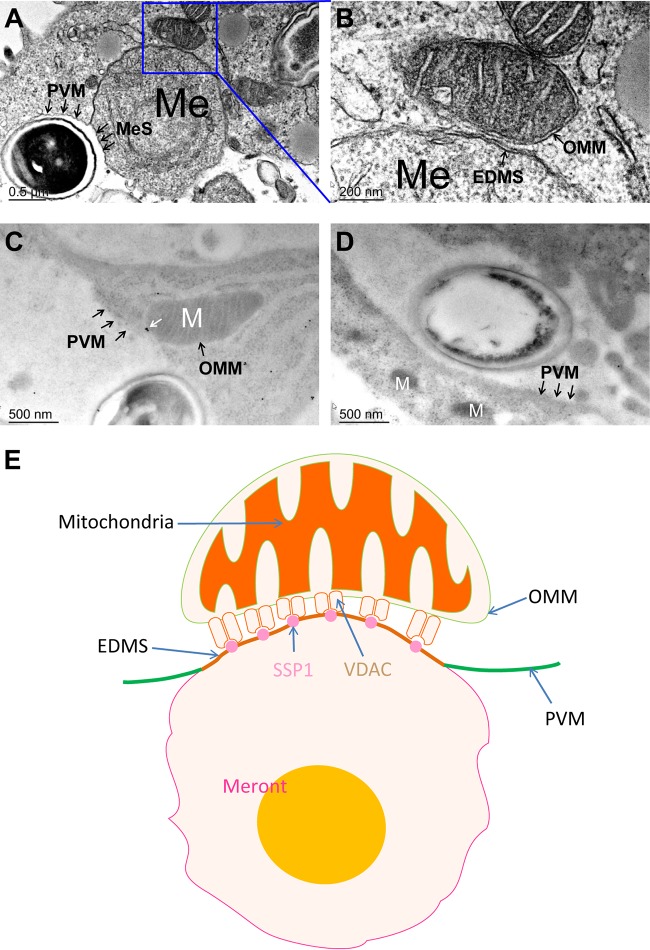
Host mitochondria interact with the PVM and meronts, and VDAC maps to this interacting interface. TEM images demonstrate host mitochondrion PV interactions. (A and B) Both panel A and enlarged panel B show clustering of mitochondria (M) and meronts (ME) and a direct interaction of the OMM with the membrane structure and PVM merging into the EDMS. (C) Immuno-EM data demonstrating that the VDACs (white arrow, gold particles) were concentrated at the site of interaction of OMM and PVM. This is consistent with a role for VDAC in parasite host mitochondrial interactions at the PVM. (D) Negative control for immune-EM. (E) Model of the binding of mitochondria with the parasitophorous vacuole via the interaction of VDAC on the OMM with SSP1 on the EDMS. Images were created using a Motifolio anatomy drawing toolkit. Bar, 0.5 μm (A) or 200 nm (B) or 500 nm (C and D).

As demonstrated in Fig. 8A, the meront surface (MeS) appears to merge with the PVM to form an EDMS. This provides a location where EhSSP1 on the meront surface would be exposed to the cytoplasm and would be able to interact with VDAC on the OMM. The enlarged image in Fig. 8B demonstrates that the OMM is in intimate contact with this EDMS. Immuno-EM performed with anti-VDAC antibody demonstrated that VDAC was concentrated at the interface of OMM and PVM (Fig. 8C). No staining was seen in the negative control (Fig. 8D). This suggests that an interaction of EhSSP1 on meronts with VDACs on the OMM is important for the association of host mitochondria with the PV. A model of this interaction is shown in Fig. 8E, with SSP1 exposed on the surface of EDMS and mitochondria binding to the PVM via the interaction of the VDAC on the OMM with SSP1.

### Silencing VDAC1, VDAC2, and VDAC3 expression by siRNA inhibits microsporidian infection and host mitochondrion association.

**FIG 9:**
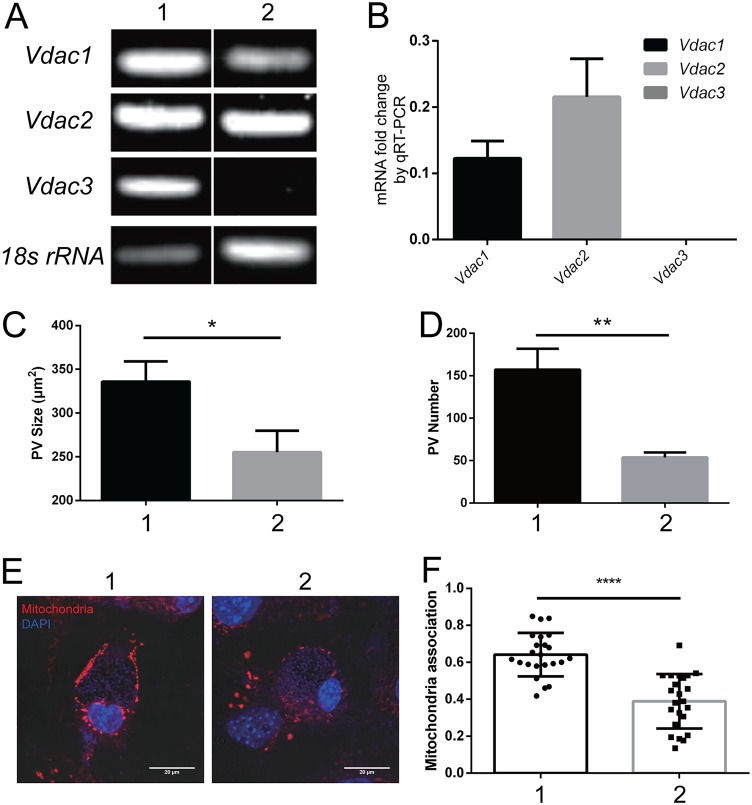
Impact of host cell *vdac1*/*2*/*3* knockdown on microsporidian infection and host mitochondrial PV association. (A) RT-PCR of *vdac1*, *vdac2*, and *vdac3* mRNA levels. Lane 1, MEF-WT plus control siRNA; lane 2, MEF-vdac1/3^−/−^ plus vdac2 siRNA. (B) qRT-PCR quantification of *vdac1*/*2*/*3* mRNA expression in MEF-vdac1/3^−/−^ plus vdac2 siRNA normalized to MEF-WT plus control siRNA cells. (C) PV size at 2 dpi in infected MEF-WT plus control siRNA and MEFvdac1/3^−/−^ plus vdac2 siRNA cells; *, *P* < 0.05. (D) Number of PV at 2 dpi in infected MEF-WT plus control siRNA and MEF-vdac1/3^−/−^ plus vdac2 siRNA cells; **, *P* < 0.01. (E) Mitochondrial staining with MitoTracker (red) in infected cell types: MEF-WT plus control siRNA and MEF-vdac1/3^−/−^ plus vdac2 siRNA. Cell nuclei were stained by DAPI (blue). Bar, 20 μm. (F) Quantification of host mitochondrial association with the PV membrane in infected MEF-WT plus control siRNA and MEF-vdac1/3^−/−^ plus vdac2 siRNA cells; ****, *P* < 0.0001 (*n* = 23 vacuoles).

To further investigate the role of the interaction of VDACs and SSP1 in microsporidian infection, we evaluated the effect of modulating VDAC expression in host cells on the ability of microsporidia to infect these cells. VDAC1, VDAC2, and VDAC3 knockdown cells were produced by treating VDAC1/3^−/−^ cells (51) with a short interfering RNA (siRNA) against *Vdac2.* The level of mRNA expression of each gene was quantified by PCR (Fig. 9A and B), with results that demonstrated an ∼88% reduction in *Vdac1* mRNA, an ∼78% reduction in *Vdac2* mRNA, and an ∼100% reduction in *Vdac3* mRNA compared to wild-type MEF (MEF-WT) cells treated with a negative-control siRNA. Microsporidian infectivity was investigated by quantifying the size and number of PVs at 2 days postinfection. As shown in Fig. 9C and D, both the size and number of PVs in infected VDAC knockdown MEF cells (lacking the VDAC1, VDAC2, and VDAC3 isoforms) were significantly reduced compared to MEF-WT cells. Host mitochondrion association was investigated using MitoTracker staining and demonstrated that the amount of mitochondria associated (i.e., binding) with the PVs was much smaller in the infected VDAC knockdown MEF cells than in the infected MEF-WT control cells (Fig. 9E and F).

## DISCUSSION

The sporoplasm is delivered by the polar tube into the invasion synapse and represents the life cycle stage that establishes the initial microsporidian infection in the host cell. It is a difficult stage to work with since it is very sensitive to osmotic stress and there are no efficient protocols to purify sporoplasms. To date, only a few proteins have been characterized in this life cycle stage (52, 53), and there is a limited amount of information on its structure and composition. In the spore, the polar tube is connected at the anterior end and then coils around the sporoplasm. After polar tube germination, the sporoplasm flows through the polar tube, appears as a droplet at the distal end of the polar tube, and remains attached to the polar tube for several minutes (54–57). It seems that the polar tube is tightly associated with sporoplasm both inside and outside the spore. On the basis of this association, we examined our cross-linked PTP extraction preparation for novel sporoplasm surface proteins that we reasoned might be involved in interactions of both the host cell and the polar tube with the sporoplasm. The cross-linked PTP extraction preparation contained PTP2, PTP3, and PTP4, which is consistent with our previous data indicating that the original DTT-solubilized PTP preparation contained components of the polar tube (23). In addition, we identified EnP1 in this preparation, and this protein was found in the anchoring disk region where the polar tube everts (13, 14). This was consistent with the concept that our cross-linked PTP preparation would contain proteins interacting with the polar tube. Examination and characterization of the most abundant unknown hypothetical proteins, in this cross-linked PTP preparation, that contained a signal peptide resulted in the identification of EhSSP1.

In addition to its interaction with the polar tube, EhSSP1 bound to the host cell surface and cell cytoplasm and, interestingly, also was able to interact with the PVM in infected cells. This binding was not random but instead reflected specific protein-protein interactions. As shown by the yeast two-hybrid experiments, EhSSP1 bound to the polar tube by interacting with PTP4. Polar tube protein 4 (PTP4) is found on the entire polar tube and has a unique epitope exposed at the anterior end of polar tube recognized by a specific anti-PTP4 monoclonal antibody (23). The interaction between EhSSP1 and PTP4 might be important as an anchor that helps to keep the sporoplasm attached to the end of polar tube after the spore germination. In addition, an interaction between EhSSP1 and PTP4 may be involved in the ability of the polar tube to coil around the sporoplasm within the intact spore, thereby establishing an anchor point for an interaction of the polar tube with the sporoplasm membrane.

The interaction of microsporidian proteins with host cell proteins is likely important for successful invasion. Several spore wall (SW) proteins (EnP1, SWP7, SWP9, SWP11, and SWP26) have been shown to bind host cells and to play important roles during the attachment of a spore to host cells (14, 58–60). This binding keeps the spore in proximity to the host cell during invasion. As components of the specific invasion apparatus of microsporidia, the polar tube proteins play a crucial role in infection. Mannosylated PTP1 can interact with unknown host cell mannose-binding molecules, resulting in adherence of the polar tube to its host cell (20, 25). The host TfR1 serves as a receptor for EhPTP4 and helps facilitate entry of the sporoplasm into the host cell, perhaps by stimulating the endocytosis pathway (23). Homologs of EhSSP1 were highly conserved in E. cuniculi, E. intestinalis, and E. romaleae, with sequence identity higher than 85% (see Fig. S1 in the supplemental material). Such SSP1 homologs were also identified by BLAST in the other microsporidian genomes in MicrosporidiaDb (www.microsporidiadb.org); however the sequence identity for homologs in these more distant microsporidia was ∼35% (Fig. S1). EhSSP1 was predicted by BLAST to share some homology with SWP7, a previously described spore wall protein in Nosema bombycis, but showed low (∼32%) sequence identity to this protein (Fig. S1). The IFA and TEM data indicated that EhSSP1 was not a spore wall protein but a membrane protein found on the surface of the sporoplasm and meronts (proliferative forms). It also was expressed in more mature life cycle stages but beneath the spore wall, where it was inaccessible to antibody for EhSSP1 or to other binding partners outside the organism.

10.1128/mBio.01944-19.1 FIG S1 Multiple-sequence alignment of EhSSP1 and homologs. The homologs of EhSSP1 in genus *Encephalitozoon* were highly conserved, with the sequence identity higher than 85%, while EhSSP1 shares low (less than 35%) sequence identity with its homologous proteins in other microsporidian species. EhSSP1, Encephalitozoon hellem SSP1, accession number EHEL_111090; E. romaleae, Encephalitozoon romaleae hypothetical protein, accession number EROM_111090; E. cuniculi, Encephalitozoon cuniculi hypothetical protein, accession number ECU11_1210; E. intestinalis, Encephalitozoon intestinalis hypothetical protein, accession number Eint_111090; O. colligata, Ordospora colligata hypothetical protein, accession number M896_121080; H. magnivora, Hamiltosporidium magnivora hypothetical protein, accession number CWI36_0708p0020; N. bombycis, Nosema bombycis hypothetical spore wall protein 7, accession number EF683107.1; N. apis, Nosema apis ABC-type multidrug transport ATPase and permease component, accession number EQB61147.1; T. ratisbonensis, Tubulinosema ratisbonensis spore wall 7 protein, accession number RVD93187.1; Ent. hepatopenaei, Enterocytozoon hepatopenaei SWP7, accession number OQS55031.1; A. algerae, Anncaliia algerae hypothetical protein, accession number H312_01036. Download FIG S1, TIF file, 2.1 MB. Copyright © 2019 Han et al. 2019 Han et al. This content is distributed under the terms of the Creative Commons Attribution 4.0 International license.

rEhSSP1 was demonstrated to bind to the surface of host cells and to the site where the tip of polar tube attached to the host cell (Fig. 4B), suggesting that it may be involved in the interaction of the host cell and sporoplasm within the invasion synapse during invasion. The PVM was formed by the host cell plasma membrane during invasion (32, 61), so those unknown targets of EhSSP1 might be incorporated into the PVM during the formation of the PV because rEhSSP1 could bind to PVM. Binding of EhSSP1 to the PVM may be involved in the localization of meronts to the internal PVM surface, while the absence of this interaction in the more mature stages expressing a spore wall may allow these stages to locate to the middle of the PV away from the PVM.

rEhSSP1, as well as antibody to EhSSP1, significantly reduced microsporidian infectivity when added to tissue culture, suggesting a role for EhSSP1 in invasion. We believe that the interaction of EhSSP1 with the host cell surface occurs in the invasion synapse when the sporoplasm is extruded from the polar tube. The ELISA data demonstrated that EhSSP1 bound both to the cell surface and to a structure within the host cell. Furthermore, greater binding was seen within the host cell, suggesting that an interacting host cell protein was more abundantly expressed within the host cell. The identification of VDAC as a host protein interacting with EhSSP1 is consistent with this observation.

VDAC proteins are also known as mitochondrial porins and are mainly expressed in the cytoplasm on the OMM (49). In addition, VDACs are expressed on plasma membranes but at lower levels (62, 63). VDAC, a major OMM protein, confers a sieve-like structure to the OMM due to its high abundance, covering ∼30% of the membrane surface (64, 65). There are three isoforms of VDAC (VDAC1, VDAC2, and VDAC3) identified in mammals, and all of these isoforms exhibit ample similarity in both sequence and structure (66). Generally, the physiologic function of VDACs is to control the movement of adenine nucleotides, NADH, and other metabolites across the outer membrane and they possess a pathological function as mediators of mitochondrion-dependent cell death through formation of the permeability pore (67–70). More recently, host cell VDACs have been reported to interact with intracellular pathogens. VDAC was identified as a component of the Mycobacterium avium phagosome membrane and was used by the bacterium for the transport of mycobacterial lipids into the cytoplasm via an interaction with mmpL4 (mycobacterial membrane protein large 4) lipoproteins (71). During infection with E. cuniculi, host cell VDAC located to points of contact between the PV and host mitochondria and was involved in scavenging ATP directly from the host (38). In this study, we demonstrated that EhSSP1, a microsporidium sporoplasm surface protein, interacts with the host cell VDAC. Our TEM data also demonstrated that VDAC was concentrated at the interface of the OMM and PVM, that mitochondria aggregated near meronts on the PVM, and that mitochondria bound to the PVM at specific areas where meronts resided in close proximity to the OMM. Furthermore, microsporidia showed a significant reduction in infectivity when infecting MEF cells lacked all three VDAC isoforms (VDAC1, VDAC2, and VDAC3) and mitochondrial association with the PV was also significantly decreased when all three VDAC isoforms were knocked down. We believe that EhSSP1 is similar in function to Toxoplasma gondii MAF1 (43) and is involved in recruiting mitochondria during PV development by interaction directly with VDAC on the OMM.

**FIG 10:**
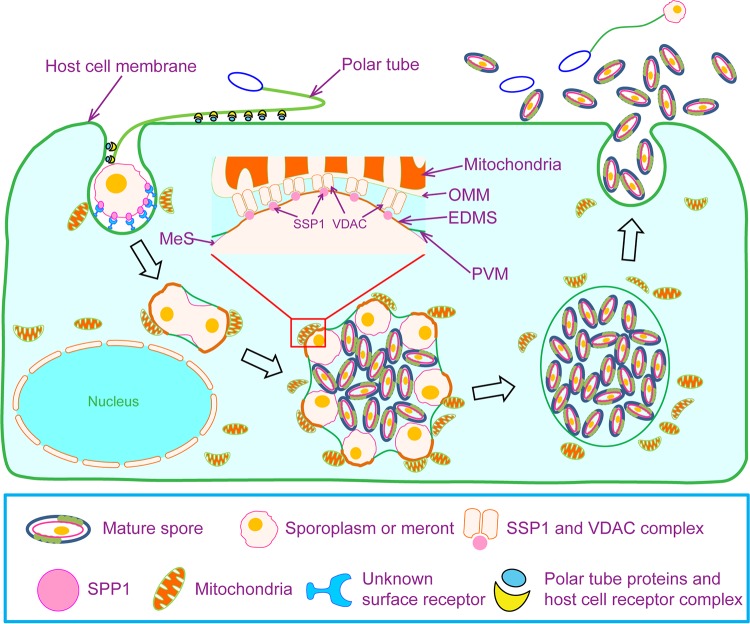
A model of microsporidian and host cell interaction during cell infection and development. After germination of the spore, the tip of polar tube enters the host cell via interactions of PTP1 and PTP4 with host cell receptors, forming an invasion synapse. The sporoplasm is then transported into this invasion synapse via the hollow polar tube. The sporoplasm surface protein SSP1 interacts with cell surface receptors (currently unknown, but possibly VDAC) and maintains sporoplasm attachment to the cytoplasm membrane, which next transforms into the PVM during invasion. As the invasion synapse is pinched off from cytoplasm membrane, it transforms into the PV as the sporoplasm becomes a meront and starts replicating. At this early infection stage, the host mitochondria are already tethering around the PVM. Within the PV, meronts attach to the PVM and subsequently begin to bulge out from the PV. The meront surface (MeS) then interacts with the PVM, forming an EDMS, which we believe is a strategy that microsporidia use to expose the meront SSP1 to the host cell cytosol. The host cell mitochondria cluster around these meronts and contact the EDMS directly. This interaction probably plays a crucial role in host cell mitochondrion associations with the PVM and in microsporidian energy acquisition from the host for meronts during replication. Images were created using a Motifolio anatomy drawing toolkit.

On the basis of previous reports and results of this study, we have proposed a model for the role of EhSSP1 in microsporidian invasion and replication (Fig. 10). According to this model, after the germination of the spore, the tip of polar tube enters the host cell via interactions of PTP1 and PTP4 with host cell receptors forming an invasion synapse, allowing transport of the sporoplasm into this invasion synapse by the hollow polar tube (23, 72). Sporoplasm surface protein 1 then interacts with cell surface receptors (currently unknown, but possibly VDACs) and helps anchor the sporoplasm to the host cell plasma membrane which becomes the PVM during the invasion process (32). As the invasion synapse is pinched off from cytoplasm membrane, it becomes the PV and the sporoplasm starts to replicate as the proliferative life cycle-stage (meronts). By this early infection stage, the host mitochondria have been already tethering around the PVM. Within the PV, the meronts attach to the PVM and bulge out from the PV. The meront surface then interacts with the PVM, forming an EDMS, which we believe is a strategy that microsporidia use to expose the meront SSP1 to the host cell cytosol. The host cell mitochondria cluster around these meronts and have direct contact with the EDMS. The interaction between the OMM protein VDAC and the EhSSP1 most likely plays a crucial role in host cell mitochondria associating with the PVM and in microsporidian energy acquisition from the host mitochondria for meronts during replication. With the formation of the spore wall, the developing microsporidian spores detach from the PVM, as SSP1 is now no longer available to interact with the PVM after being buried under the spore wall, and the mature spores eventually move to the middle of the PV. Eventually, the PV lyses and mature spores are released into the environment to start the infective cycle anew.

Overall, the data in this paper support the model that microsporidia bind to host mitochondria by hijacking VDAC using EhSSP1, which is probably critical for energy (ATP) uptake by the replicative forms of this organism. Further work is ongoing on the identification of other proteins that interact with EhSSP1 and of other sporoplasm surface proteins using the methods described in this paper. Understanding how microsporidia mediate host cell interactions will provide insight into the biology of this pathogen and may serve to provide new therapeutic targets for microsporidiosis.

## MATERIALS AND METHODS

### Cells and reagents.

RK13 (rabbit kidney) cells (ATCC, CCL-37) were cultured in 10% fetal bovine serum (FBS) (Thermo Fisher, Waltham, MA) minimum essential medium (MEM) with penicillin-streptomycin at 5% CO_2_. Human foreskin fibroblasts (HFF) (ATCC, CRL-2522) and mouse embryonic fibroblasts (MEF-WT and VDAC1/3^−/−^ MEFs [51]; gift of Frank Edlich, University of Freiburg) were maintained in Dulbecco’s modified essential medium (DMEM) with penicillin-streptomycin supplemented with 10% FBS at 5% CO_2_. Anti-VDAC1 rabbit polyclonal antibody (catalog no. PA1-954A; Invitrogen, Carlsbad, CA) was used for IFA. Horseradish peroxidase (HRP)-conjugated anti-mouse secondary antibodies, Alexa Fluor 488-conjugated goat anti-mouse secondary antibody, and Alexa Fluor 594-conjugated goat anti-rabbit secondary antibodies were purchased from Thermo Fisher Scientific. Rabbit anti-EcPTP1 polyclonal antibody used for IFA and correlative fluorescence and scanning electron microscopy (CLEM) was produced previously in our laboratory (73). All the buffered solutions were purchased from Sigma-Aldrich (St. Louis, MO). All reagents used are commercially available, and the chemicals used were of the highest analytical grade available from the supplier.

### Encephalitozoon hellem culture.

RK13 (rabbit kidney) cells were maintained in 10% FBS–MEM–penicillin-streptomycin at 5% CO_2_. Confluent monolayers were infected with E. hellem spores. The spores were collected from culture media, purified by passing them through a 5-μm-pore-size filter (Millipore, Billerica, MA) to remove host cells, concentrated at 6,000 × *g* by centrifugation at room temperature for 10 min, and stored in sterile distilled water at 4°C. Spores used in these experiments were counted with a hemocytometer (three times for each sample and then averaged).

### Protein purification and liquid chromatography-electrospray ionization-tandem mass spectrometry (LC-ESI-MS/MS) analysis.

Five hundred million purified E. hellem spores were harvested, spun down at 16,500 × *g* for 5 min at room temperature, and then disrupted in 1% SDS lysis buffer containing protease inhibitor (protease inhibitor cocktail) using 0.4 g of 0.5-μm-diameter acid-washed glass beads (Sigma-Aldrich) in a Mini-BeadBeater (BioSpec Products, Bartlesville, OK) for 3 cycles of 1 min per cycle. The disrupted spore suspension was then clarified by centrifugation at 16,500 × *g* for 10 min, and the pellet was resuspended in reaction buffer containing 100 mM HEPES, 600 mM NaCl, and 4 mM DSP (thiol-cleavable dithio-bissuccinimidylpropionate; Sigma-Aldrich); incubated for 30 min at 4°C; and then quenched with 40 mM lysine (22). The tube was then centrifuged, and the pellet was washed five times with 1% SDS and then once with 9 M urea. The pellet was then solubilized in 2% DTT for 2 h at room temperature, the tube was centrifuged, and the supernatant was collected for proteomics analysis. A 50-μl volume of 2× protein sample buffer (0.5 M Tris-HCl [pH 6.8], 4.4% SDS, 20% glycerol, 2% 2-mercaptoethanol, 0.01% bromophenol blue) was mixed with the same volume of supernatant, and the mixture was boiled for 5 min. The sample was then centrifuged, and the supernatant was separated using SDS-PAGE followed by staining of the gel with Coomassie brilliant blue and analysis performed using our previously reported protocol (23). Briefly, an excised gel band was reduced, alkylated, and digested with trypsin. LC-ESI-MS/MS analysis of the peptide digests was then performed by C_18_–reversed-phase chromatography using an UltiMate 3000 RSLCnano system (Thermo Fisher Scientific) equipped with an Acclaim PepMap rapid-separation liquid chromatography (RSLC) C_18_ column (Thermo Fisher Scientific) (2-μm pore size, 100 Å, 75 μm by 15 cm). The ultra-high-performance liquid chromatography (UHPLC) system was connected to a TriVersa NanoMate nanoelectrospray source (Advion, Ithaca, NY) and a linear ion trap LTQ-XL (Thermo Scientific) mass spectrometer with an ESI source operated in the positive-ionization mode. The Mascot generic format (MGF) files generated from the raw LC-ESI-MS/MS data were searched by Mascot (version 2.5.1; Matrix Science, Boston, MA) against the NCBInr90_20141124 database (25,782,812 protein sequences) with the following search parameters: trypsin; two missed cleavages; peptide charges of +2 and +3; peptide tolerance of 1.5 Da; MS/MS tolerance of 0.5 Da; carbamidomethylation (Cys) for fixed modification; deamidation (Asn and Gln) and oxidation (Met) for variable modifications. A decoy database search was also performed to measure the false-discovery rate.

For immunoprecipitation, HFF cells were grown to confluence in a T25 flask, washed gently once with phosphate-buffered saline (PBS), and incubated with rEhSSP1–PBS for 1 h at 4°C. Bovine serum albumin (BSA) was used as a negative control. Then cells were washed with PBS three times and lysed in 1 ml radioimmunoprecipitation assay (RIPA) buffer (Thermo Fisher Scientific catalog no. 89900) with EDTA-free protease inhibitor cocktail (Roche, Germany; catalog no. 11836170001) for 20 min at 4°C. The cell lysate was spun down at 16,500 × *g* for 5 min at 4°C and the supernatant collected. Fifty microliters of anti-HA magnetic beads (Pierce, Waltham, MA; catalog no. 88836) were incubated in PBS with 2 μg of rat anti-HA-tagged monoclonal antibody (clone 3F10) (Sigma-Aldrich catalog no. 11867423001) at 4°C for 1 h. The anti-HA magnetic bead conjugates were then washed three times with PBS and resuspended in a final volume of 500 μl cell lysate prepared as noted above. The samples were then incubated at 4°C for 2 h with gentle shaking followed by separation of the beads on a magnetic platform, and then the immune system pellets were washed three times with PBS. The samples were then boiled in 2× protein sample buffer and centrifuged, and the supernatants were separated on an SDS-PAGE gel. The gel was then stained with either Coomassie brilliant blue or silver using standard methods. LC-MS/MS analysis was conducted as described above.

### Cloning, protein expression, and purification of EhSSP1.

The coding region of EhSSP1 was PCR amplified from E. hellem genomic DNA using Q5 High-Fidelity DNA polymerase (New England Biolabs, Ipswich, MA) with specific primers (see Table S2 in the supplemental material) and ligated into a pET32a-derived expression vector with a 3× HA tag added to the N terminus of the recombinant protein. The resulting plasmid was transformed into BL21(DE3) cells (Sigma-Aldrich). Transformed bacteria were grown in Luria broth (LB) containing ampicillin (100 μg/μl) until an optical density at 600 nm (OD_600_) of 0.6 to 0.8 nm was reached. Expression of the recombinant proteins was then induced with 0.2 mM isopropyl-β-d-1thiogalactopyranoside (Sigma) for another 20 h at 30°C before harvesting. The bacterial cells were pelleted and resuspended in buffer A (20 mM Tris-HCl [pH 7.5], 200 mM NaCl). The cells were lysed using an EmulsiFlex C3 homogenizer (Avestin, Ottawa, Canada), and separation of the lysate from the intact cells was achieved by centrifugation (16,500 × *g*, 30 min). The supernatant was loaded onto a HisPure nickel-nitrilotriacetic acid (Ni-NTA) column (Thermo Fisher Scientific) that had been equilibrated previously with buffer A. The column was then successively washed with 10 column volumes (CV) of buffer A followed by 10 CV of buffer B (20 mM Tris-HCl [pH 7.5], 200 mM NaCl, 50 mM imidazole). The protein was then eluted with 5 CV of buffer C (20 mM Tris-HCl [pH 7.5], 200 mM NaCl, 500 mM imidazole). Protein purity was determined by SDS-PAGE, and protein concentrations were determined using a NanoDrop spectrophotometer (Thermo Fisher Scientific).

10.1128/mBio.01944-19.7 TABLE S2 List of primers used in this study. Download Table S2, DOC file, 0.04 MB. Copyright © 2019 Han et al. 2019 Han et al. This content is distributed under the terms of the Creative Commons Attribution 4.0 International license.

### Expression of EhSSP1 in mammalian cells.

The coding region of EhSSP1 was PCR amplified from E. hellem genomic DNA using Q5 High-Fidelity DNA polymerase (New England Biolabs) with specific primers (Table S2) and ligated into a pcDNA3.1 vector (Invitrogen, San Diego, CA) that contained the human cytomegalovirus (CMV) immediate early promoter (pCMV) and a 3× HA tag added to the N terminus. The vector construct was then transfected into HFF cells using Lipofectamine 3000 reagent (Thermo Fisher Scientific catalog no. L3000001). A mock transfection was also prepared with an empty pcDNA 3.1 vector. HFF cells grown to 70% to 90% confluence in six-well plates in fresh medium (DMEM with 10% FBS and 1% penicillin-streptomycin). The transfection was conducted according to the manufacturer’s instructions. Protein expression was detected by IFA 48 h after transfection.

### Antibody production.

For preparation of a polyclonal murine antiserum, 500 μg of purified rEhSSP1 protein was injected into five BALB/c mice after emulsification with Freund’s complete adjuvant. Each mouse received 100 μg of rEhSSP1. After boosts administered monthly for 3 months, the mice were bled and their sera were collected for use.

Polyclonal rabbit antibody to rEhSSP1 was produced by Harlan Laboratories (Envigo, Indianapolis, IN) following their standard 112-day polyclonal antibody protocol. Briefly, a New Zealand White rabbit was initially immunized with 200 μg of purified rEhSSP1 emulsified with Freund’s complete adjuvant followed by three monthly injections of 100 μg of purified rEhSSP1 emulsified with Freund’s incomplete adjuvant. The ELISA titer of the rabbit serum was assessed following each boost injection and was >1:50,000 1 month following the final injection. Rabbit serum (rPcAb-EhSSP1) was collected 1 month following the final immunization and stored at –20°C. Preimmunization rabbit serum was collected and screened to confirm the absence of endogenous antibody that reacted with E. hellem or EhSSP1. Preimmunization rabbit serum stored at –20°C was used as a negative control for experiments using polyclonal rabbit antisera.

### Electron microscopy.

For transmission electron microscopy (TEM), RK13 cells were grown to confluence in six-well plates and then infected with 1 × 10^6^ spores for 3 days. Cells were fixed with 2.5% glutaraldehyde–2% paraformaldehyde–0.1 M sodium cacodylate buffer, postfixed with 1% osmium tetroxide followed by 2% uranyl acetate, dehydrated through a series of ethanol gradients, and embedded in LX112 resin (Ladd Research Industries, Burlington, VT). Ultrathin sections were cut on a Reichert Ultracut UCT ultramicrotome, stained with uranyl acetate followed by lead citrate, and viewed on a JEOL 1400EX transmission electron microscope at 80 kV.

For immunoelectron microscopy (immuno-EM), infected cells were fixed with 4% paraformaldehyde–0.05% glutaraldehyde–0.1 M sodium cacodylate buffer, dehydrated through a series of ethanol gradients, with a progressive lowering of the temperature to –50°C, in a Leica EMAFS (Electron Microscopy Automatic Free Substitution) system, embedded in Lowicryl HM-20 monostep resin (Electron Microscopy Sciences, Hatfield, PA), and polymerized using UV light. Ultrathin sections were cut on a Reichert Ultracut E ultramicrotome, immunolabeled with antibodies of interest, and then stained with uranyl acetate followed by lead citrate. Stained sections were viewed on a JEOL 1400EX transmission electron microscope at 80 kV.

### Correlative fluorescence and scanning electron microscopy (CLEM).

One million suspended spores were aspirated and pipetted on glass coverslips coated with 0.01% poly-l-lysine, and the spores were then germinated by incubation with germination buffer (140 mM NaCl, 1 mM CaCl_2_, 1 mM MgCl_2_, 5 mM KCl, pH 7.5) for 15 min at room temperature. A final concentration of 5% H_2_O_2_ was added to the germination buffer, and the spores were incubated for another 15 min at room temperature. Germinated spores were fixed with 4% paraformaldehyde–PBS for 30 min at 37°C. After being washed three times with Tris-buffered saline (TBS) containing 0.05% Tween 20 (TBST), coverslips were blocked by the use of 3% BSA–TBST for 1 h at room temperature. EhSSP1 mouse polyclonal antibody (1:100 dilution) and EcPTP1 rabbit polyclonal antibody (1:500 dilution) were incubated with spores for 1 h at room temperature. After the reaction mixture was washed three times with TBST, Alexa Fluor 488 fluorescein isothiocyanate-labeled anti-mouse IgG antibody and Alexa Fluor 594 fluorescein isothiocyanate-labeled anti-rabbit IgG antibody were each added at a 1:500 dilution. Samples were washed three times with TBST and imaged using a Zeiss Axio Observer microscope equipped with AxioVision software with the “shuttle & find” setting to mark cell locations. After fluorescence imaging, samples were fixed with 2.5% glutaraldehyde, dehydrated in ethanol, subjected to critical point drying (Tousimis Samdri 790), and coated with chromium (EMS 150T-ES). The same cells were automatically located in a Zeiss Supra 40 field emission scanning electron microscope and imaged with a secondary electron detector. Fluorescence and scanning electron microscope images were correlated by the use of Zeiss AxioVision software.

### Binding ELISA.

To detect binding on fixed permeabilized cells, HFF cells were grown to confluence in 96-well plates and fixed with 4% paraformaldehyde–PBS for 30 min at 37°C. The fixed cells were permeabilized with 0.1% Triton X-100 for 20 min at room temperature. Nonspecific binding sites were blocked by incubation with ELISA blocking buffer (Thermo Fisher Scientific). After washing was performed three times with TBST, serially diluted EhSSP1 soluble protein (starting from 2 μg) was added to each well for 1 h at room temperature. Washing with TBST was then performed three times to remove unbound proteins. EhSSP1 rabbit polyclonal sera were added at a 1:2,000 dilution, and the mixture was maintained for 1 h at room temperature. After being washed three times with TBST, anti-rabbit IgG alkaline phosphatase antibody was added at a 1:2,000 dilution and the plates were incubated for 1 h at room temperature. After washing was performed three times with TBST, p-nitrophenyl phosphate substrate (Sigma-Aldrich catalog no. N2640) was added and the absorbance at 405 nm was read using an MRXe microplate reader (Dynex, Chantilly, VA). BSA was used at the same serial concentration as EhSSP1 for negative-control plates.

To detect binding on live cells, HFF cells were grown to confluence in 96-well plates and washed gently once with PBS. Serial dilutions of EhSSP1 soluble protein (starting from 2 μg) were added to each well, and the mixtures were incubated 1 h at 4°C. Unbound proteins were then washed off gently three times with PBS. Cells were then fixed with 4% paraformaldehyde–PBS for 30 min at 37°C and blocked by incubation with ELISA blocking buffer. The incubation of primary and secondary antibodies and the reaction with the ELISA substrate were conducted using the protocol described above.

### Immunofluorescence assay.

Cells were fixed with 4% paraformaldehyde–PBS for 30 min at 37°C and were then subjected to permeabilization with 0.1% Triton X-100–PBS at 37°C for 20 min. The cells were washed with TBST three times and blocked with 3% BSA–TBST at 4°C overnight. All incubations with primary and secondary antibodies were done at 37°C for 1 h in a moist chamber. The dilutions of the antibodies and probes used were as follows: EhSSP1 mPcAb, 1:100; EcPTP1 rPcAb, 1:500; VDAC antibody, 1:250; MitoTracker (Invitrogen catalog no. M7512), 100 nM. Cells were washed three times with TBST and then incubated with appropriate secondary antibody and with DAPI (4′,6-diamidino-2-phenylindole) at 1:500. After incubation, the cells were washed three times with TBST and mounted with ProLong Gold antifade solution (Invitrogen). Photomicrographs were taken with either a SP5 confocal microscope (Leica, Buffalo Grove, IL) or a Microphoto-FXA epifluorescence microscope (Nikon, Melville, NY).

### Yeast two-hybrid assay.

The coding region of EhSSP1 was PCR amplified from E. hellem genomic DNA using Q5 High-Fidelity DNA polymerase (New England Biolabs) with specific primers (Table S2) and ligated into yeast two-hybrid bait vector pGBKT7-BD. In a similar fashion, the coding regions of EhPTP1, EhPTP2, EhPTP3, EhPTP4, EhPTP5, and EhSSP1 were PCR amplified from E. hellem genomic DNA and the coding regions of VDAC1, VDAC2, and VDAC3 were PCR amplified from human genomic DNA using Q5 High-Fidelity DNA polymerase (New England Biolabs) with specific primers (Table S2) for ligation into yeast two-hybrid prey vector pGADT7-AD. The bait construction and prey construction were transformed into competent Y2H Gold yeast cells and competent Y187 yeast cells, respectively. The transformed yeast cells were cultured using a high-stringency screen following the protocol of the manufacturer (Biosciences Clontech, San Jose, CA).

For interaction studies, the Y2H Gold yeast cells containing the bait construct were mated with each of the prey constructs in Y187 yeast cells according to the mating protocol of the manufacturer. The mated yeast cells were plated on QDO/X/A plates (leucine, tryptophan, histidine, and adenine quadruple dropout plates supplemented with X-α-galactosidase [X-α-Gal] and aureobasidin A). The plates were incubated at 30°C for 5 to 7 days until colonies appeared. Y2H Gold yeast cells that were transformed with pGBKT7-53 were mated with Y187 yeast cells that were transformed with pGADT7-T as the positive control, and Y2H Gold yeast cells that were transformed with pGBKT7Lam were mated with Y187 yeast cells that were transformed with pGADT7-T as the negative control.

### Infectivity competition and blocking assay.

For the protein competition assay, 1 × 10^5^ HFF cells were cultured in a 24-well plate overnight. Aliquots of purified EhSSP1 (5 μg/ml) were inoculated onto HFF cells for 1 h at room temperature. BSA (5 μg/ml) was used as a negative control. One million spores were placed onto a monolayer of HFF and incubated for 12 h until infectivity was detected. Spore invasion was detected by fluorescent *in situ* hybridization (FISH) as described in our previous publication (23). Briefly, coverslips in the 24-well plates were washed gently with PBS to remove free spores and fixed with 1:1 methanol/acetone for 10 min at room temperature, after which the fixative was removed and the coverslips were allowed to air-dry at room temperature for 30 min. Prehybridization of the coverslips was performed with 100 μl hybridization buffer (Sigma-Aldrich catalog no. 11717472001) for 1 h at 57°C in a humid chamber. Hybridization was then carried out by removing the prehybridization solution and then adding a 20 μM concentration of HEL878F probe (Alexa Fluor 594 ACTCTCACACTCACTTCAG [Thermo Fisher Scientific HPLC-purified custom oligonucleotide]) to hybridization buffer at 57°C overnight on a circular rocking platform in a humid chamber. The coverslips were then washed twice with 2× SSC (1× SSC is 0.15 M NaCl plus 0.015 M sodium citrate) for 15 min at 57°C and mounted with ProLong Gold antifade mountant with DAPI (Thermo Fisher Scientific). Coverslips were evaluated using a Nikon Microphot FXA Microscope and a triple-band D/F/TR filter cube (XF467; Omega Optical, Brattleboro, VT) to determine infectivity rates. Twenty high-power (×40) fields (HPFs) were counted for quantification of red (intracellular) forms and of the number of HFF cells in each HPF to determine percent invasion at 12 h postinfection. Only red intracellular forms were counted as invaded cells. Any spores that had both red and blue staining were not counted as these were interpreted to represent spores that were either present very early in invasion or unable to correctly invade the host cells. Blue-staining spores were extracellular and also not counted. Invasion rates were expressed as percent invasion ± standard error of the means (SEM).

For the antibody blocking assay, 1 × 10^5^ HFF cells were cultured in a 24-well plate overnight. E. hellem spores were incubated with EhSSP1 PcAb (∼5 μg/ml) for 1 h at room temperature and then this mixture was added to the HFF monolayers. Negative-testing mouse or rabbit sera were used as control antibodies. One million spores were placed onto a monolayer of HFF and incubated for 12 h, and the invasion was detected by fluorescent *in situ* hybridization (FISH) as described above.

### *Vdac2* silencing by siRNA in MEF-VDAC1/3^−/−^ cells.

The MEF-WT and MEF-VDAC1/3^−/−^ cells were seeded onto 24-well plates (1 × 10^5^ cells/well) and cultured overnight to a confluence of 70%. MEF cells were then transfected with 5 nM *Vdac2* siRNA (Invitrogen catalog no. 4390771, identifier [ID] s75924) or a nontargeting siRNA (Silencer Select negative-control no. 1; Invitrogen catalog no. 4390843) using Lipofectamine RNAiMAX transfection reagent (Invitrogen catalog no. 13778030) according to the manufacturer’s instructions. To confirm the expression of VDAC1, VDAC2, and VDAC3, total RNA was isolated using a Qiagen RNeasy minikit (Qiagen, Germantown, MD; catalog no. 74134) and quantified using a NanoDrop ND-1000 spectrophotometer (NanoDrop Technologies, Inc.). RNA was subjected to reverse transcription (RT) into cDNA using a One*Taq* RT-PCR kit (New England BioLabs catalog no. E5310S). Forward and reverse primers for reverse transcription-quantitative PCR (qRT-PCR) are summarized in Table S3.

10.1128/mBio.01944-19.8 TABLE S3 List of primers for qRT-PCR. Download Table S3, DOC file, 0.03 MB. Copyright © 2019 Han et al. 2019 Han et al. This content is distributed under the terms of the Creative Commons Attribution 4.0 International license.

### Quantification of microsporidian infection and host mitochondria PV association in host cells.

At 1 day after the MEF cells were transfected with siRNA, fresh purified E. hellem spores were added to the MEF cell culture (1 × 10^6^ spores/well) to infect the MEFs. To quantify the E. hellem infection 2 days postinfection, the cultures were washed 3 times with PBS and then fixed with 4% paraformaldehyde. Spores in PVs were stained by the use of 0.01% Fluorescent Brightener 28 (Sigma-Aldrich catalog no. F3543) for 10 min at room temperature. Photomicrographs of 20 fields of each well were randomly taken at ×20 using a Microphoto-FXA epifluorescence microscope (Nikon), and quantification of the size and number of PV was performed using ImageJ.

To quantify the host mitochondrion association, infected MEF cells were stained with 100 nM MitoTracker mixed in incomplete DMEM for 30 min and then fixed with 4% paraformaldehyde. Cell nuclei were stained by the use of DAPI (1:1,000 dilution). Photomicrographs of 23 parasitophorous vacuoles from each group were randomly taken with a SP5 confocal microscope (Leica), and mitochondrion association was quantified by measuring the percentage of the PV membrane surrounded by host mitochondria using ImageJ.

### Ethics statement.

All animal experiments were conducted according to the guidelines of the U.S. Public Health Service Policy on Humane Care and Use of Laboratory Animals. Animals were maintained in an AAALAC-approved facility, and all protocols were approved by the Institutional Care Committee of the Albert Einstein College of Medicine, Bronx, NY (Animal Protocol no. 20180605, 20180606, 20180607, and 20180608; Animal Welfare Assurance no. A3312-01). No human samples were used in these experiments. Human foreskin fibroblasts (HFF) were obtained from ATCC.

### Statistics.

Experiments were performed in triplicate and repeated at least three times. Each experiment was performed separately with its own negative control. The significance of differences between the results from control and experimental assays was evaluated using a two-tailed Student's *t* test. *P* values of 0.05 or less were considered statistically significant. *P* values of 0.01 or less were considered highly significant. Data were also analyzed using the Mann-Whitney U test (nonparametric statistics), and the results confirmed the degree of significance seen with the two-tailed Student's *t* test.

### Data availability.

The sequence of E. hellem SSP1 is present in the GenBank database under accession number EHEL_111090.
