# Supplementary material for: Microsporidia Interact with Host Cell Mitochondria via Voltage-Dependent Anion Channels Using Sporoplasm Surface Protein 1
Source: mBio. 2019 Aug 20;10(4):e01944-19. doi: 10.1128/mBio.01944-19 (PMC6703431; doi:10.1128/mBio.01944-19)
Supplement: TABLE S1 [file mBio.01944-19-st001.docx]

Table S1 Hypothetical proteins identified from LC-MS/MS analysis

|  | MicrosporidiaDB ID | GenBank Access ID | Product Description | Mw (kDa) | Signal Peptide |
| --- | --- | --- | --- | --- | --- |
| 1 | KMI_081970 | EHEL_081970 | hypothetical protein | 29.05 | Yes |
| 2 | KMI_050080 | EHEL_050160 | hypothetical protein | 36.59 | Yes |
| 3 | KMI_050580 | EHEL_050660 | hypothetical protein | 27.23 | Yes |
| 4 | KMI_010880 | EHEL_010950 | hypothetical protein | 43.60 | Yes |
| 5 | KMI_111040 | EHEL_111090 | SPP1 (this study) | 32.99 | Yes |
| 6 | KMI_020070 | EHEL_020090 | hypothetical protein | 24.66 | Yes |
| 7 | KMI_071370 | EHEL_071390 | hypothetical protein | 71.02 | Yes |
| 8 | KMI_081900 | EHEL_081900 | hypothetical protein | 41.93 | Yes |
| 9 | KMI_051060 | EHEL_051140 | hypothetical protein | 29.45 | Yes |
| 10 | KMI_010230 | EHEL_010300 | hypothetical protein | 15.87 | Yes |
| 11 | KMI_020190 | EHEL_020210 | hypothetical protein | 15.79 | Yes |
| 12 | KMI_071510 | EHEL_071530 | hypothetical protein | 55.01 | Yes |
| 13 | KMI_091380 | EHEL_091340 | hypothetical protein | 16.58 | Yes |
| 14 | KMI_031420 | EHEL_031440 | hypothetical protein | 14.86 | Yes |
| 15 | KMI_041490 | EHEL_041510 | hypothetical protein | 22.62 | Yes |
| 16 | KMI_111140 | EHEL_111190 | hypothetical protein | 45.57 | Yes |
| 17 | KMI_040080 | EHEL_040100 | hypothetical protein | 37.58 | Yes |
| 18 | KMI_070880 | EHEL_070900 | hypothetical protein | 35.29 | Yes |
| 19 | KMI_010620 | EHEL_010690 | spore wall and anchoring disk complex protein EnP1 | 39.46 | Yes |
| 20 | KMI_060140 | EHEL_060160 | Polar tube protein 2 | 29.21 | Yes |
| 21 | KMI_111280 | EHEL_111330 | Polar tube protein 3 | 138.50 | Yes |
| 22 | KMI_071060 | EHEL_071080 | Polar tube protein 4 | 31.79 | Yes |
