# Supplementary figures and images for: Microsporidia Interact with Host Cell Mitochondria via Voltage-Dependent Anion Channels Using Sporoplasm Surface Protein 1
Source: mBio. 2019 Aug 20;10(4):e01944-19. doi: 10.1128/mBio.01944-19 (PMC6703431; doi:10.1128/mBio.01944-19)

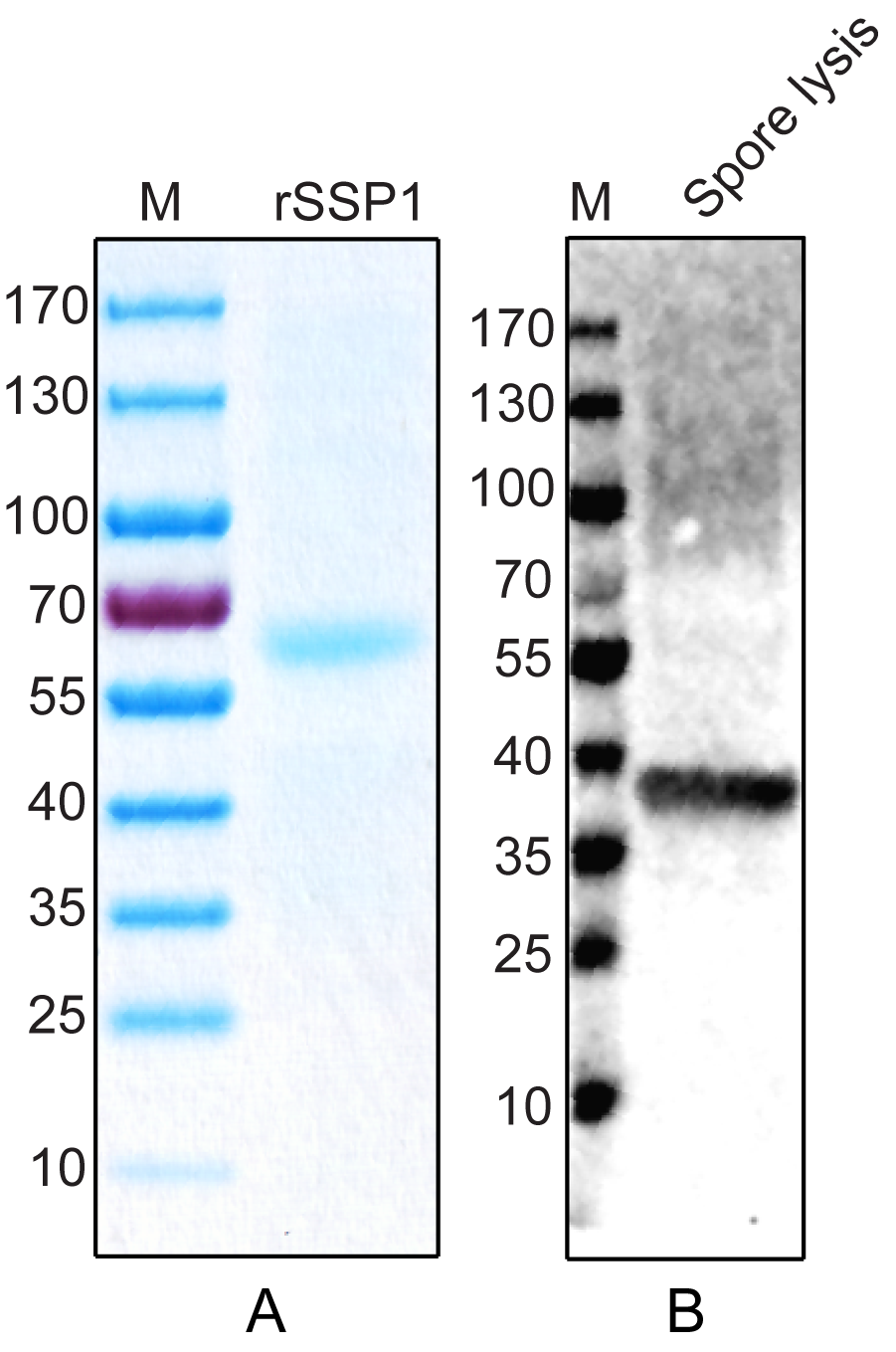

Supplement: FIG S2 [file mBio.01944-19-sf002.tif]

EhSSP1 mPcAb

HA-tag mAb

Merge

Merge&amp;Phase

rEhSSP1

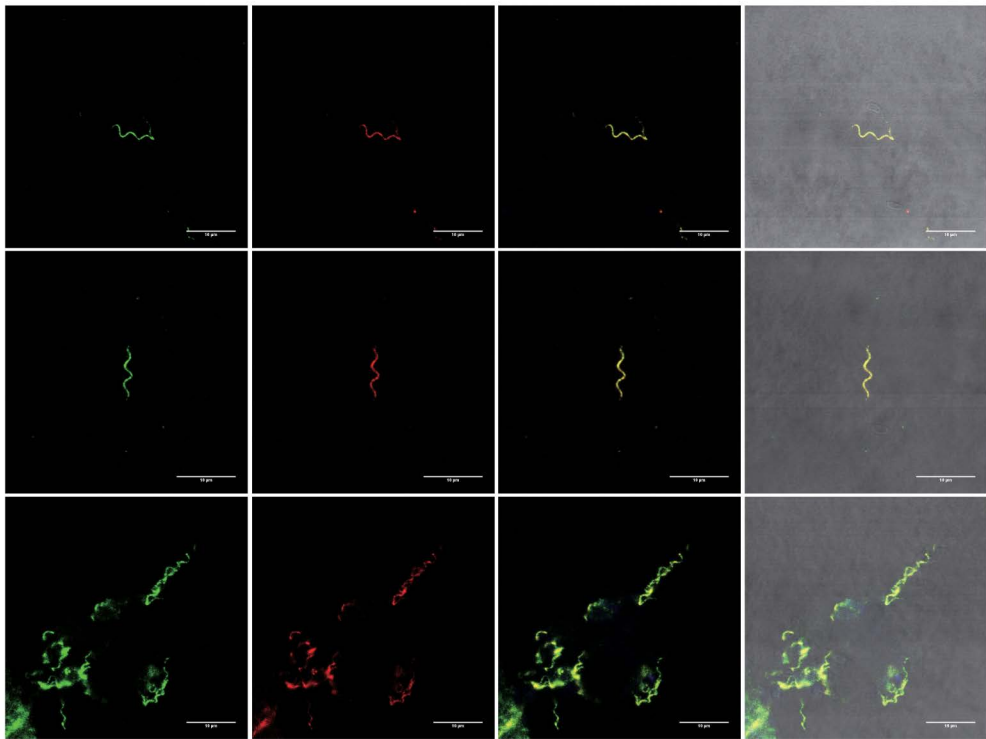

Control

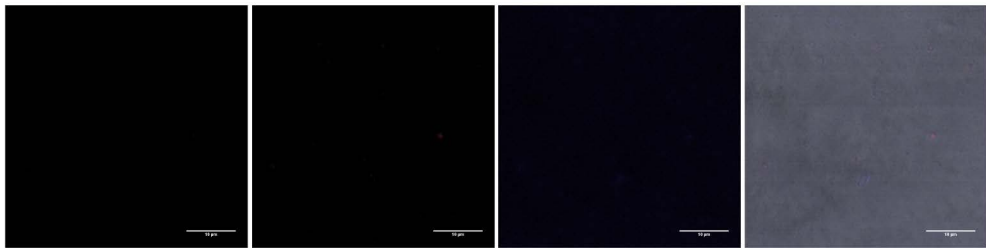

Supplement: FIG S3 [file mBio.01944-19-sf003.pdf]

rEhSSP1

HA

Merge

Merge&amp;Phase

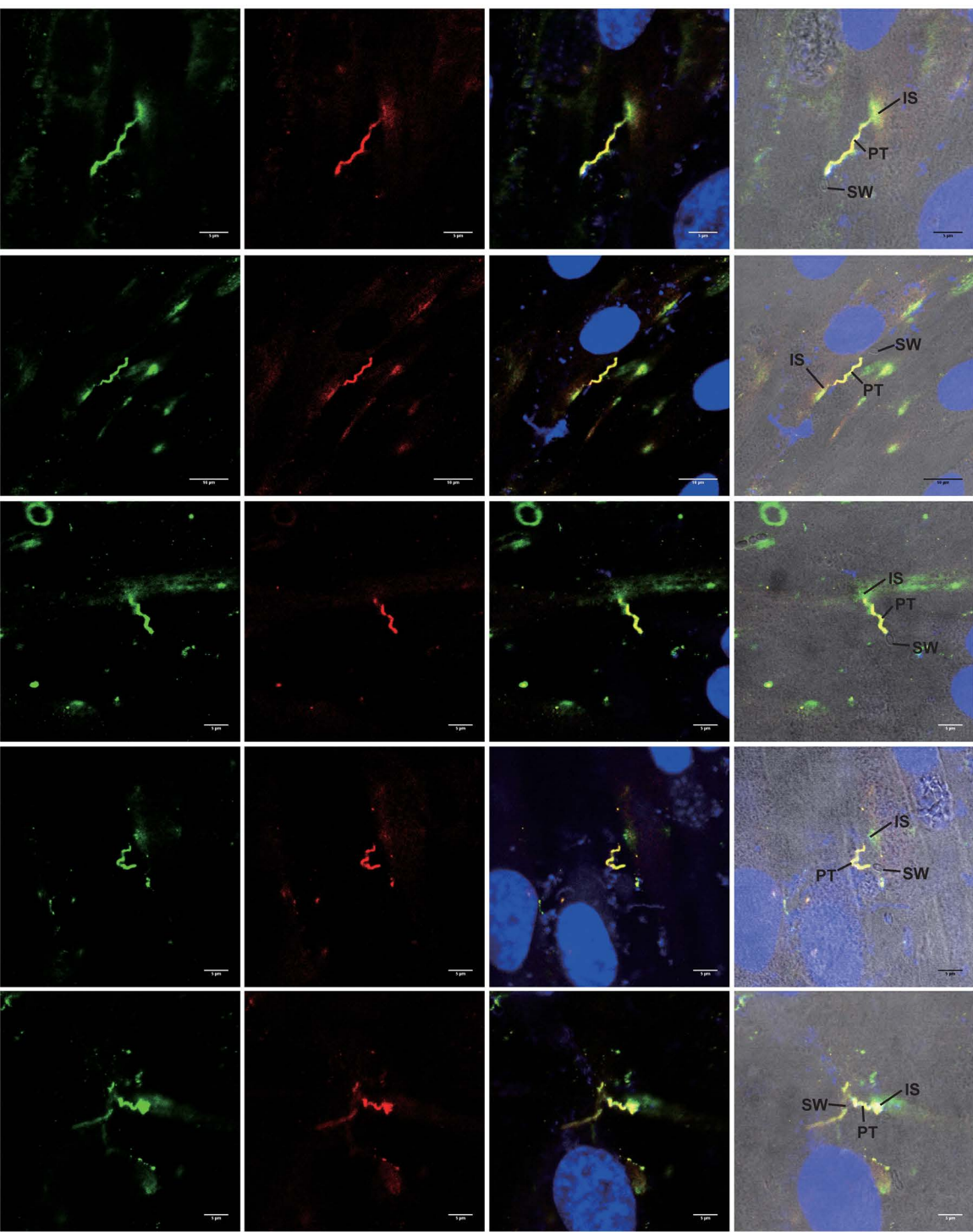

Supplement: FIG S4 [file mBio.01944-19-sf004.pdf]

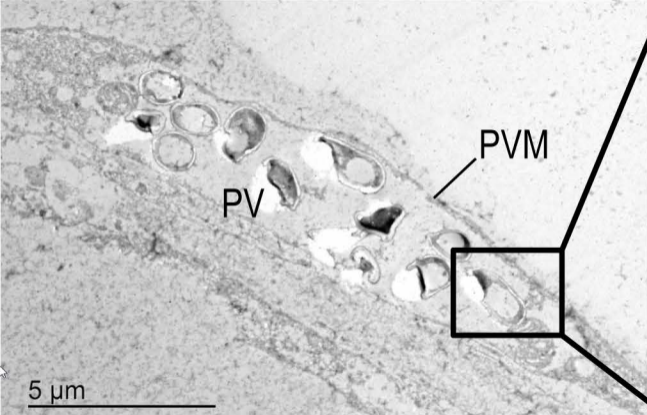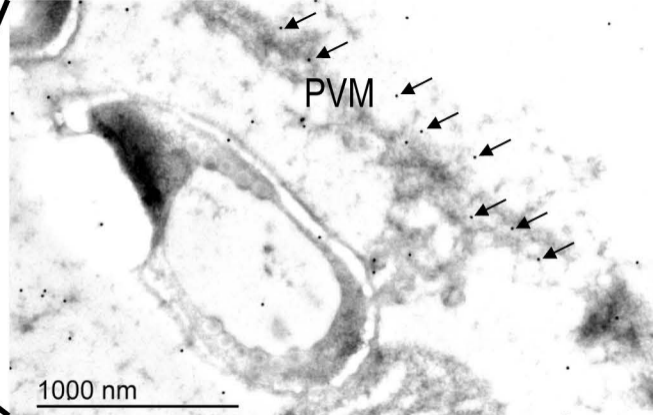

Supplement: FIG S5 [file mBio.01944-19-sf005.pdf]

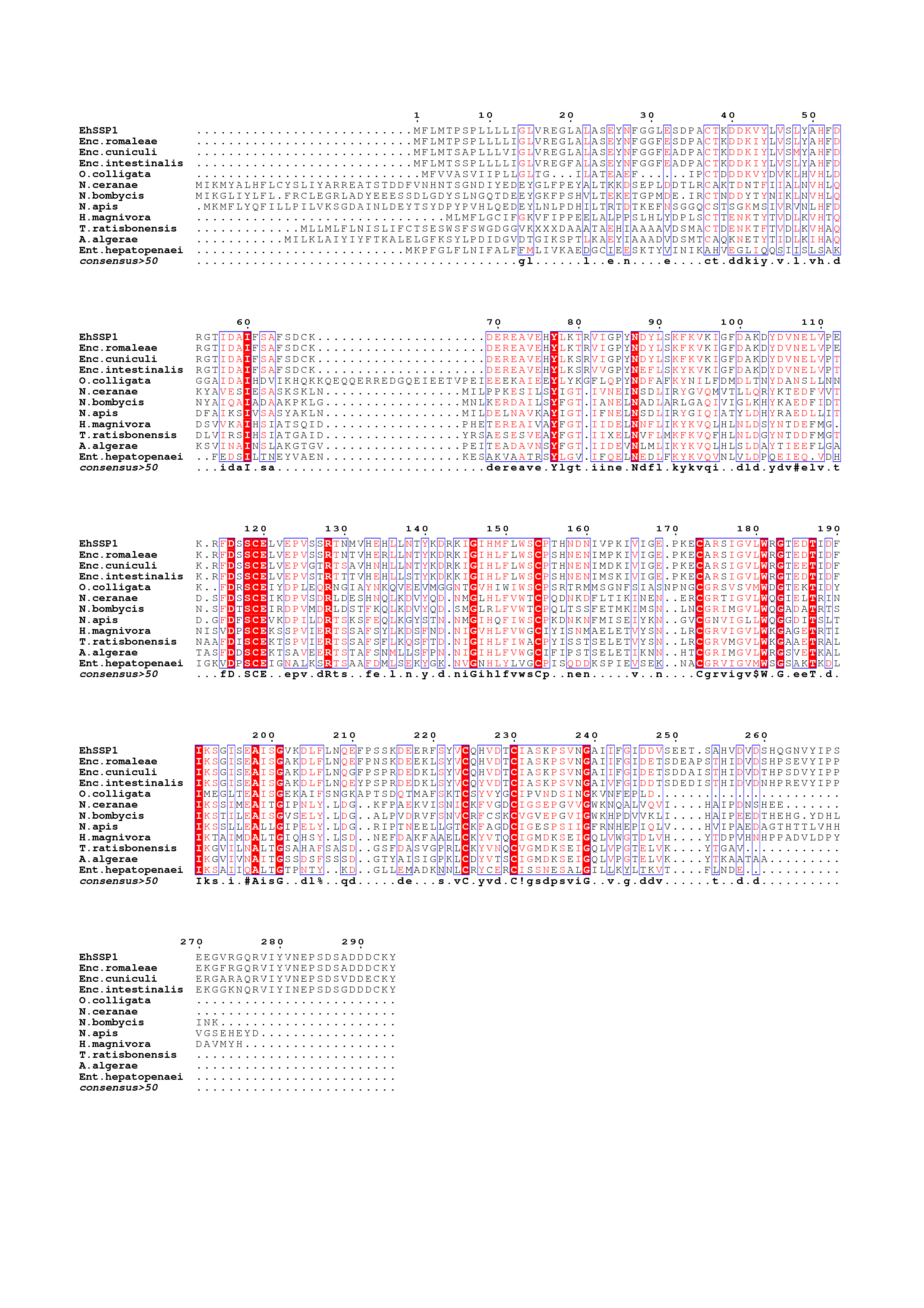

Supplement: FIG S1 [file mBio.01944-19-sf001.tif]
