# Supplementary material for: Microsporidia Interact with Host Cell Mitochondria via Voltage-Dependent Anion Channels Using Sporoplasm Surface Protein 1
Source: mBio. 2019 Aug 20;10(4):e01944-19. doi: 10.1128/mBio.01944-19 (PMC6703431; doi:10.1128/mBio.01944-19)
Supplement: TABLE S2 [file mBio.01944-19-st002.doc]

**Table S2. List of primers used in this study**

| Primers | Sequences of oligonucleotides（5’→3’） |
| --- | --- |
| pET32a-HA-Forward | TACCCGTACGACGTCCCGGACTACGCTAGCCCGGGCTTCTCCTCAAC |
| pET32a-HA-Reverse | CTTGTCGTCGTCATCGGTACC |
| pET32a-EhSSP1-Forward | GTACCGATGACGACGACAAGTCTGAATATAACTTCGGAG |
| pET32a-EhSSP1-Reverse | TCCGGGACGTCGTACGGGTAGTACTTGCAGTCATCGTC |
| pcDNA-Forward: | TACCCGTACGACGTCCCG |
| pcDNA-Reverse | GGTGGCTAGCCAGCTTG |
| pcDNA-EhSSP1-Forward: | ACCCAAGCTGGCTAGCCACCATGTTTCTCATGACACCATC |
| pcDNA-EhSSP1-Reverse | TCCGGGACGTCGTACGGGTAGTACTTGCAGTCATCGTCG |
| pGBKT7-Forward | ATCCGTCGACCTGCAGCGGC |
| pGBKT7-Reverse | GAATTCGGCCTCCATGGCCA |
| pGADT7-Forward | CATCGATACGGGATCCATCG |
| pGADT7-Reverse | ACCCGGGTGGAATTCACTGG |
| pGBKT7-BD-EhSSP1-F | TGGCCATGGAGGCCGAATTCTCTGAATATAACTTCGGAGG |
| pGBKT7-BD-EhSSP1-R: | GCCGCTGCAGGTCGACGGATTTAGTACTTGCAGTCATCGT |
| pGADT7-AD-VDAC1-F | CCAGTGAATTCCACCCGGGTATGGCTGTGCCACCCA |
| pGADT7-AD-VDAC1-R | GATCCCGTATCGATGTTATGCTTGAAATTCCAGTCCTAGA |
| pGADT7-AD-VDAC2 -F | CCAGTGAATTCCACCCGGGTATGGCGACCCACGGACAGA |
| pGADT7-AD-VDAC2-R | CGATGGATCCCGTATCGATGTTAAGCCTCCAACTCCAGGG |
| pGADT7-AD-VDAC3-F | CCAGTGAATTCCACCCGGGTATGTGTAACACACCAACGTA |
| pGADT7-AD-VDAC3-R | CGATGGATCCCGTATCGATGTTAAGCTTCCAGTTCAAATC |
