# Supplementary material for: Microsporidia Interact with Host Cell Mitochondria via Voltage-Dependent Anion Channels Using Sporoplasm Surface Protein 1
Source: mBio. 2019 Aug 20;10(4):e01944-19. doi: 10.1128/mBio.01944-19 (PMC6703431; doi:10.1128/mBio.01944-19)
Supplement: TABLE S3 [file mBio.01944-19-st003.doc]

**Table S3. List of primers for qRT-PCR**

| Primers | Sequences of oligonucleotides（5’→3’） |
| --- | --- |
| Mouse 18S  ribosomal RNA (r18S)-F: | CTTTCGAGGCCCTGTAATTG |
| Mouse 18S  ribosomal RNA (r18S)-R: | CCTCCAATGGATCCTCGTTA |
| Mouse VDAC1-F: | AAGTGAACAACTCTAGCCTGA |
| Mouse VDAC1-R: | CACCCGCATTGACGTTCTTG |
| Mouse VDAC2-F: | CCAAGTCAAAGCTGACAAGGA |
| Mouse VDAC2-R: | TTTAGCTGCAATGCCAAAAC |
| Mouse VDAC3-F: | TTGACACAGCCAAATCCAAA |
| Mouse VDAC3-R: | TGTTGCTGCCAGCTGTCCAT |
